# Template-Free Assembly of Functional RNAs by Loop-Closing Ligation

**DOI:** 10.1021/jacs.2c05601

**Published:** 2022-07-26

**Authors:** Long-Fei Wu, Ziwei Liu, Samuel J. Roberts, Meng Su, Jack W. Szostak, John D. Sutherland

**Affiliations:** † MRC Laboratory of Molecular Biology, Francis Crick Avenue, Cambridge Biomedical Campus, Cambridge CB2 0QH, United Kingdom; ‡ Department of Molecular Biology and Center for Computational and Integrative Biology, Howard Hughes Medical Institute, Massachusetts General Hospital, Boston, Massachusetts 02114, United States; § Department of Genetics, Harvard Medical School, Boston, Massachusetts 02115, United States; ∥ Department of Chemistry and Chemical Biology, Harvard University, Cambridge, Massachusetts 02138, United States

## Abstract

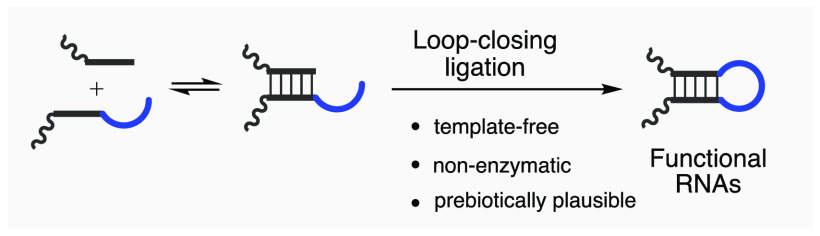

The first ribozymes are thought to have emerged at a time when RNA replication proceeded via nonenzymatic template copying processes. However, functional RNAs have stable folded structures, and such structures are much more difficult to copy than short unstructured RNAs. How can these conflicting requirements be reconciled? Also, how can the inhibition of ribozyme function by complementary template strands be avoided or minimized? Here, we show that short RNA duplexes with single-stranded overhangs can be converted into RNA stem loops by nonenzymatic cross-strand ligation. We then show that loop-closing ligation reactions enable the assembly of full-length functional ribozymes without any external template. Thus, one can envisage a potential pathway whereby structurally complex functional RNAs could have formed at an early stage of evolution when protocell genomes might have consisted only of collections of short replicating oligonucleotides.

## Introduction

**Figure 1:**
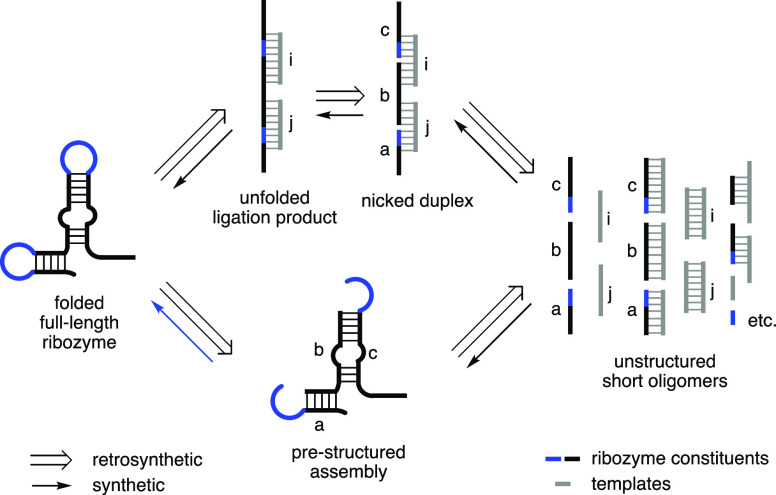
Potential loop-closing ligation constructs stem-loop hairpin structure directly in a template-free manner. Conventional nicked duplex ligation strategy (top pathway) and the potential loop-closing ligation strategy (bottom pathway) to assemble structured, full-length functional RNAs from short oligonucleotides.

Functional RNAs such as ribozymes, riboswitches, and aptamers—both naturally occurring and those selected by in vitro evolution—adopt folded structures.^1−5^ The prebiotic generation of structured RNA is thus crucial to the emergence of functional ribozymes during the origin of life. However, the compact, folded structure required for catalysis is incompatible with the demand for an unstructured RNA as a template for copying. Thus, a fragmentation strategy has been widely explored to assemble full-length RNAs nonenzymatically, based on the rationale that copying the unstructured, constituent fragments individually would be less problematic than copying the structured, full-length strands. The emergence of functional RNAs can be divided into two stages: first, the copying and replication of the short fragments^6−8^ and then an assembly process leading to functional RNAs.^9,10^ Two strategies for the assembly process have been explored. The template-directed ligation of adjacent oligonucleotides was first reported in 1966^11^ and has been studied in many contexts in subsequent decades.^12−16^ The advantage of this strategy is that it allows many ways to position ligation junctions and template strands (nicked duplex in Figure 1, top pathway) so as to assemble the unfolded form of the structured ribozyme (unfolded ligation product in Figure 1, top pathway). However, ribozymes assembled in this manner are inevitably subject to inhibition by a full-length template or even by shorter splint templates.^7−10,16^ Either product purification to remove the template^9^ or the design of splints that bind strongly enough to allow for ligation but weakly enough to avoid inhibition^10^ are needed to enable RNA function. Previous efforts to assemble ribozymes by splinted ligation also made use of 3′-amino terminated^10^ or 2′,3′-aminoacylated oligonucleotides^17^ to increase the efficiency of ligation. A further problem with the generation of complex-structured RNAs is that the final structure is only realized during the folding stage (unfolded ligation product in Figure 1, top pathway). In extant biology, chaperones can guide the folding of functional RNAs,^18,19^ but absent chaperones, proper folding of complex RNAs is usually imperfect, with a substantial fraction of molecules becoming trapped in metastable mis-folded states.^20^

An alternative strategy leaves the fragments noncovalently joined, dividing a full-length functional RNA into shorter pieces by interrupting the RNA chain within loops (prestructured assembly in Figure 1, bottom pathway). This strategy was pioneered by Doudna et al. three decades ago^21^ and has subsequently been explored for many other ribozymes and for aptamers.^22−24^ The noncovalently assembled complexes generated in this way are typically less active than an uninterrupted full-length strand and often exhibit greater sensitivity to conditions such as low fragment concentration and elevated temperature.^21^ Inspired by our newly discovered nicked loop acyl transfer chemistry^25^ (Figure S1), we reasoned those nicked loops might be nonenzymatically sealed because of the physical proximity of the termini of a nicked but pre-organized loop. If this could be done without disrupting a multifragment ribozyme assembly (Figure 1, bottom pathway), it would offer a strategy to directly assemble full-length structured RNAs from short oligonucleotides. Such an approach could potentially circumvent template inhibition, misfolding, and disassembly issues simultaneously. Here, we demonstrate the assembly of full-length RNA structures containing hairpin stem loops, without the need for any external template to guide the assembly process.

## Results

### Establishing Loop-Closing Ligation

**Figure 2:**
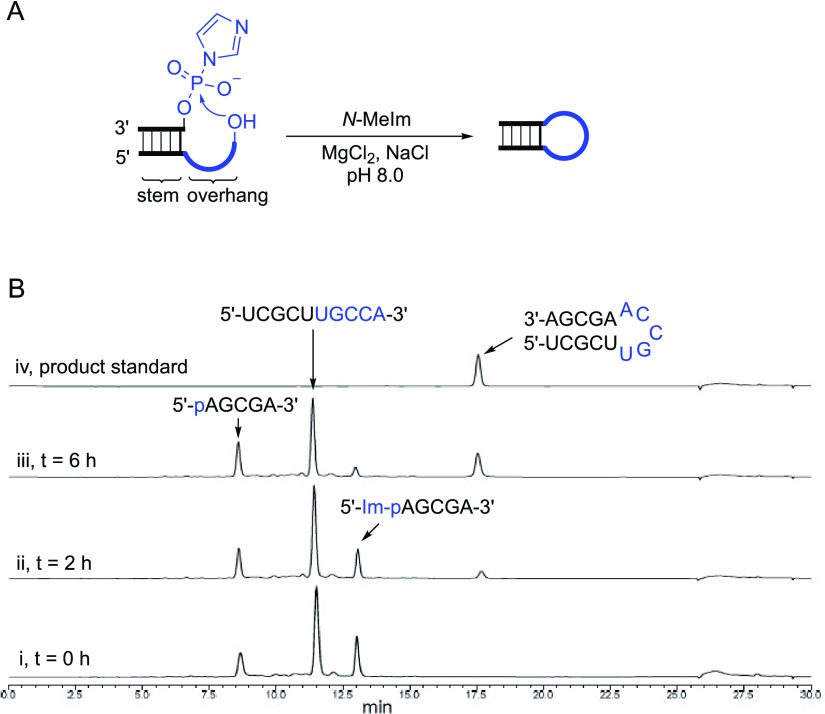
Loop-closing ligation constructs an RNA hairpin stem-loop structure directly. (A) Reaction scheme of loop-closing ligation reaction. Overhang sequence and hairpin loop region are highlighted in blue. Standard reaction conditions: phosphate donor strand in total 50 μM, including 5′-p-RNA and 5′-Im-p-RNA, phosphate acceptor strand 50 μM, MgCl_2_ (50 mM), NaCl (200 mM), *N*-methylimidazole (*N*-MeIm, 50 mM), HEPES (50 mM), pH 8.0 at 20 °C. (B) Representative time course of a loop-closing reaction monitored by HPLC at 260 nm UV detection. Peaks of RNA substrates and loop-closing product are indicated. Product standard was obtained by solid-phase RNA synthesis.

To test the feasibility of loop-closing ligation, we prepared a 5′-phosphorimidazolide-activated RNA oligonucleotide (Im-p-AGCGA-3′) and annealed it with a partially complementary 10-mer RNA with a 3′-overhang (5′-UCGCUUGCCA-3′, complementary sequence underlined, Figure 2A). After 7 days, all of the initially activated oligonucleotide had either been hydrolyzed to unactivated material or had been converted to a new product with the same retention time on high-performance liquid chromatography (HPLC) as a synthetic standard of the expected 15-mer stem-loop product of loop-closing ligation. The observed yield of 9% based on total pentanucleotide corresponds to a corrected yield, based upon the percentage of Im-p-AGCGA-3′ at the beginning of the reaction, of 16%. The observed rate of consumption of Im-p-AGCGA (0.02 h^–1^ with reaction half-life 40 h, Figure S2, Table S1) is the sum of the of first-order rates of loop-closing ligation and competing hydrolysis. Based on the partition of activated oligonucleotide between the stem-loop product (16%) and hydrolysis (84%), the actual rate of loop-closing ligation was 0.006 h^–1^, which is comparable with previously reported rates for nicked duplex ligation using 5′-phosphorimidazolide RNAs.^15^

To increase the reaction rate, we added *N*-methylimidazole (*N*-MeIm), a nucleophilic catalyst^26^ (for mechanism see Figure S3), to the above reaction and observed a concentration-dependent increase in the reaction rate. The reaction half-life decreased from 40 to 0.6 h as the concentration of *N*-MeIm increased from 0 to 200 mM, while ligation yields remained almost constant as expected from similar catalysis of ligation and hydrolysis (Table S1). After systematically exploring other parameters including temperature, pH, concentration of MgCl_2_, and concentration of NaCl that affect the model reaction (Tables S1–S4), a standard condition for loop-closing ligation (50 mM *N*-MeIm, 50 mM MgCl_2_, 200 mM NaCl, and 50 mM HEPES, pH 8.0 at 20 °C) was chosen, in which the reactions proceed to completion in less than 10 h (Figures 2B and S2).

### Exploring the Scope of Loop-Closing Ligation

Given the variety of hairpin loops that are present in functional RNAs,^27−30^ we wished to explore the scope of the loop-closing ligation. Using the overhang UGCCA as a reference sequence (30% corrected yield, Entry 1, Table 1, Figure S4), we investigated the effect of varying the length and sequence of the 3′-overhang. Shortening the overhang length to 2 or 3 nucleotides resulted in very poor ligation yields (⩽5% corrected, Entries 1–4, Table 1, Figures S5–S7), while lengthening the overhang to 6-nucleotides also decreased the corrected yield (Entry 1 versus Entry 7, Table 1, Figure S8). Presumably, a 2- or 3-nucleotide long overhang cannot easily adopt a folded conformation that brings the 5′ and 3′ termini into proximity, while the entropic cost of correctly structuring a longer overhang is increased.^28^ Changes to the sequence of 4 or 5 nucleotide overhangs significantly affect the yield of loop-closing ligation. For example, a UCCA overhang gave a three-fold higher yield than an ACCA overhang (Entries 5 and 6, Table 1, Figures S9 and S10); interestingly, this same effect was previously observed for nicked loop acyl transfer.^25^ In contrast, changing the G at the second position of the original 5-nucleotide overhang to each of the other three nucleobases had no major effect on the ligation yield or rate (Entries 8–10, Table 1, Figures S11–S13). However, changing the 3′-terminal A into C or U greatly decreased the yield (to 4 and 2% corrected, respectively, Entries 11 and 12, Table 1, Figures S14 and S15), while a change from A to G was well tolerated (30% corrected yield, Entry 13, Table 1, Figure S16). These results suggest that the first and last nucleotides of the overhang are particularly important, with the former likely being important in allowing a U-turn conformation and the latter in stacking to the last base pair of the stem.

**Table 1:** Efficiency of Loop-Closing Ligation Depends on the Overhang Length and Sequence Identity^a^

entry	phosphate acceptor sequence^b^	observed yield^c^ (%)	corrected yield^d^ (%)	reaction half-life^e^ (*t*_1/2_, h)
1	5'-UCGCUUGCCA-3'	22	30	1.8
2	5'-UCGCU**CA**-3'	0	0	1.4
3	5'-UCGCU**CCA**-3'	1	2	1.4
4	5'-UCGCU**UCA**-3'	4	5	1.5
5	5'-UCGCU**UCCA**-3'	12	15	1.6
6	5'-UCGCU**ACCA**-3'	4	5	1.5
7	5'-UCGCU**U**GCCCA-3'	8	13	1.8
8	5'-UCGCUU**A**CCA-3'	14	19	1.7
9	5'-UCGCUU**C**CCA-3'	17	21	1.5
10	5'-UCGCUU**U**CCA-3'	22	30	2.0
11	5'-UCGCUUGCC**C**-3'	2	4	1.4
12	5'-UCGCUUGCC**U**-3'	1	2	1.4
13	5'-UCGCUUGCC**G**-3'	21	30	1.3
14	5'-UCGC**C**UGCCA-3'	5	7	1.5
15	5'-UCGCUUGCC**A(2'd)**-3'	1	2	1.9
16	5'-UCGCUUGCC**A(3'd)**-3'	<1	1	1.6

a Reaction conditions: Phosphate donor strand in total 50 μM, including 5′-p-RNA and 5′-Im-p-RNA, phosphate acceptor strand 50 μM, MgCl_2_ (50 mM), NaCl (200 mM), *N*-methylimidazole (*N*-MeIm, 50 mM), HEPES (50 mM), pH 8.0 at 20 °C for 10 h.

b Phosphate donor sequence is 5′-Im-p-AGCGA-3′ for all the reactions. Complementary sequence, forming the stem region with the donor strand, is underlined.

c Observed yield is calculated as the percentage of starting material converting to the loop-closing product based on the integration of peaks on HPLC (for details, see Methods in the Supporting Information).

d Corrected yield = Observed yield divided by the initial fraction of 5′-Im-p-AGCGA-3′ present in the presynthesized mixture of 5′-Im-p-AGCGA-3′ and 5′-p-AGCGA-3′ at the beginning of the reaction (for synthetic methods, see Methods in the Supporting Information).

e Reaction half-life, *t*_1/2_, is calculated from the combined rates of first-order consumption of Im-p-AGCGA resulting from both loop-closing ligation and competing hydrolysis. For a representative time course, see Figure S2. All yields and half-lives are average values from at least two independent experiments.

With respect to the stem, changing the closing base pair from A:U into the mismatch A:C, which shortens the stem to four base-pairs while increasing the loop to 7 nucleotides, decreased the yield to 7% (Entry 14, Table 1, Figure S17). Taken together, our observations show that the efficiency of loop-closing ligation is highly dependent on the length and sequence of the stem and the overhang. Changing the 3′-terminal ribonucleoside into either a 2′- or a 3′-deoxyribonucleoside suppressed the loop-closing ligation severely (<2% corrected yield in either case, Entries 15 and 16, Table 1, Figures S18 and S19. The effect of the sugar moiety of the 3′-terminal nucleoside might simply be explained by the lower p*K*_a_ of the diol of a ribonucleoside relative to the single alcohol of a deoxynucleoside.

Regarding the 2′-5′ versus 3′-5′ regioselectivity of the loop-closing ligation, we observed that the new phosphodiester bonds formed are predominantly 2′,5′-linked in 5 out of 7 tested examples, rather than the canonical 3′,5′-linkage, as determined by comparison to synthetic standards on RNA PAGE analysis (Figure S20). However, in two other cases, we observed predominantly 3′-5′-linked products, which suggests that the regioselectivity of loop-closing ligation is sequence-dependent. The introduction of 2′,5′-linkages into functional RNAs has previously been demonstrated to be well tolerated^31^ and will be addressed further below.

### Applying Loop-Closing Ligation to the Assembly of a tRNA Minihelix

tRNAs are ubiquitous small folded RNAs with a secondary structure consisting of three stem loops. The tRNA minihelix is a truncated tRNA molecule consisting of the tRNA acceptor stem overhang and the TΨC stem loop; it has previously been shown to be recognized and enzymatically aminoacylated by an aminoacyl-tRNA synthetase.^32^ The tRNA minihelix was originally proposed as a potential evolutionary precursor of modern tRNA.^25,33^ We asked whether loop-closing ligation could be applied to construct a tRNA minihelix from shorter oligonucleotides in a template-free manner.

**Figure 3:**
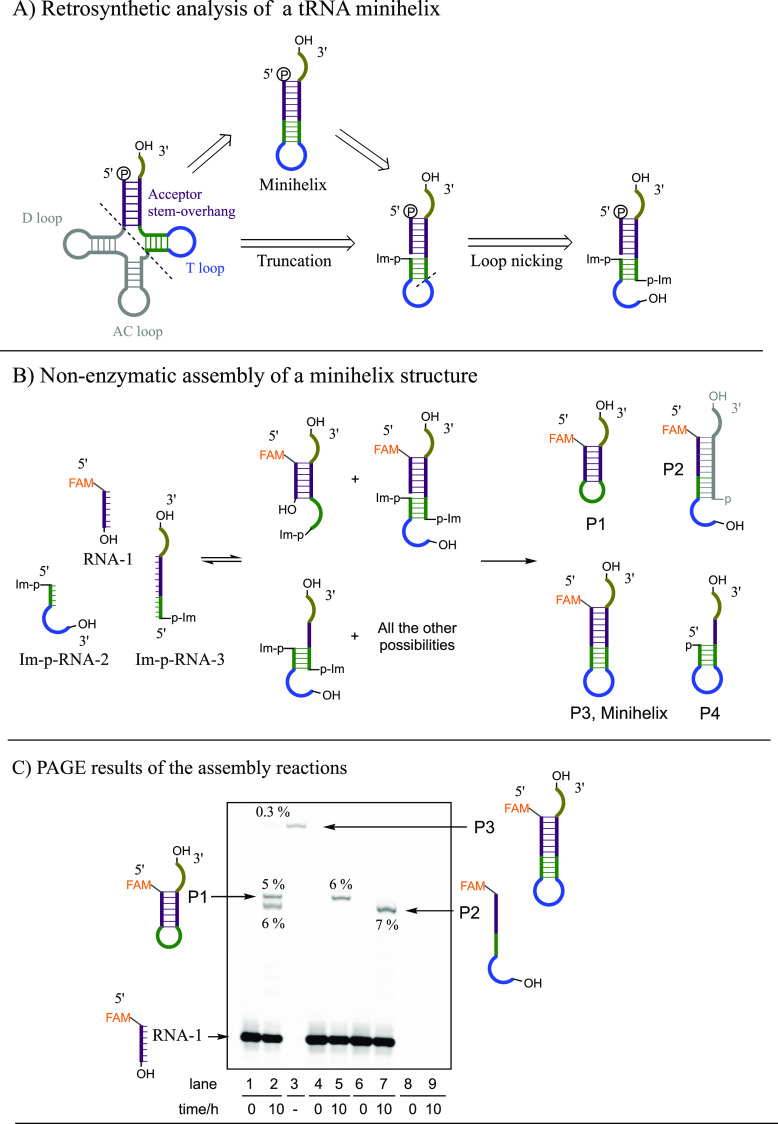
Direct assembly of a tRNA minihelix by loop-closing ligation. (A) Retrosynthetic truncation of tRNA and disconnection of the minihelix. (B) Reaction scheme for the assembly of a tRNA minihelix. Condition of full reaction: 10 uL of reaction mixture, containing RNA-1 (25 μM), Im-p-RNA-2 (25 μM in total, including Im-p-RNA-2 and p-RNA-2), Im-p-RNA-3 (25 μM in total, including Im-p-RNA-3 and p-RNA-3), NaCl (200 mM), MgCl_2_ (50 mM), *N*-methylimidazole (50 mM) in HEPES buffer (50 mM, pH 8.0), was incubated at 25 °C for 10 h. (C) Representative PAGE analysis of the assembly reactions. Lanes 1 and 2, assembly reaction of RNA-1, Im-p-RNA-2, and Im-p-RNA-3; Lane 3, authentic standard of the minihelix RNA; Lanes 4 and 5, reaction of RNA-1 and Im-p-RNA-3; Lanes 6 and 7, reaction of RNA-1, Im-p-RNA-2, and p-RNA-3; Lanes 8 and 9, reaction of p-RNA-2 and Im-p-RNA-3 (no FAM-labeled oligos in this reaction). Yields are average values observed from duplicates.

Conceptually, the tRNA minihelix can be disconnected into three fragments (Figure 3A). The RNA fragment destined to become the 5′-terminus of the tRNA minihelix (RNA-1) was 5′-FAM-labeled to enable convenient monitoring of the assembly process by gel electrophoresis. The 5′-phosphorylated fragments 2 and 3 (p-RNA-2, p-RNA-3) were converted into phosphorimidazolides (Im-p-RNA-2, Im-p-RNA-3) before mixing with RNA-1 (Figure 3B). Three FAM-labeled products (P1, P2, and P3 in observed yields of 5, 6, and 0.3%, respectively) were observed after incubating all three fragments together (each at 50 μM) under standard loop-closing ligation conditions for 10 h (Figure 3B, Lanes 1 and 2 in Figure 3C). P3 is the expected tRNA minihelix, formed by one nicked duplex ligation and one loop closing ligation, and confirmed by comparison to a standard prepared by conventional synthesis (Lane 3 in Figure 3C). P1 represents an off-target loop-closing ligation product of RNA-1 and Im-p-RNA-3, which results from the formation of a five base-pair duplex between these two oligonucleotides. The identity of P1 was confirmed by the fact that it is the only product formed when RNA-1 and Im-p-RNA-3 are incubated together (6% observed yield, Lane 4 and 5 in Figure 3C). P2 represents the on-pathway product of nicked duplex ligation between RNA-1 and Im-p-RNA-2 (templated by p-RNA-3 or Im-p-RNA-3); it is the only product observed (7% observed yield, Lanes 6 and 7 in Figure 3C) when RNA-1 and Im-p-RNA-2 are incubated in the presence of unactivated p-RNA-3 as a template. A fourth product (P4) lacking a FAM label was also expected from the loop-closing ligation reaction of Im-p-RNA-2 (or p-RNA-2) and Im-p-RNA-3. Indeed, P4 was observed when Sybr-Gold staining was used to image the RNA gel (Figure S21) and was further verified by incubating p-RNA-2 and Im-p-RNA-3 in the absence of FAM-labeled RNA-1.

These results demonstrate that loop-closing ligation can construct functional RNA structures from short oligonucleotides. Three short RNA oligonucleotides generated the desired tRNA minihelix (P3) and two on-pathway intermediate products (P2 and P4). The formation of the off-target product (P1) in the full reaction (Lanes 1&2 in Figure 3C) shows that loop-closing ligation can occur even in the presence of a short strand that is complementary to the overhang sequence. In a prebiotic scenario, our observations suggest that a pool of activated short oligonucleotides could have given rise to longer and more structurally complex RNAs than simple duplexes. To demonstrate this directly, we further tested loop-closing ligation with regard to the assembly of functional RNAs.

### Applying Loop-Closing Ligation to the Assembly of Functional Ribozymes

**Figure 4:**
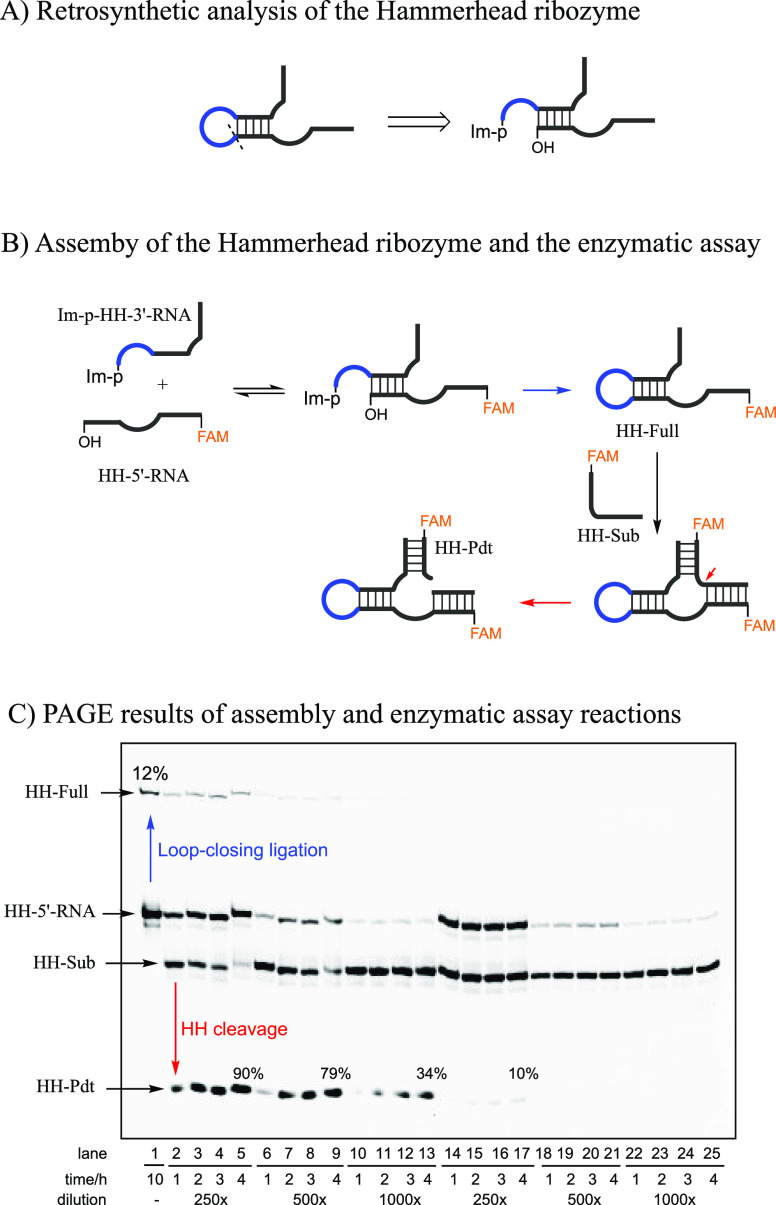
Direct assembly of the hammerhead ribozyme by loop-closing ligation. (A) Hammerhead ribozyme was disconnected at the loop region retrosynthetically. (B) Reaction scheme of the loop-closing ligation and the subsequent enzymatic cleavage of the Hammerhead substrate. Conditions for loop-closing ligation: A 10 μL reaction mixture, containing HH-5′-RNA (50 μM), Im-p-HH-3′-RNA (in total 50 μM, including Im-p-HH-3′-RNA and p-HH-3′-RNA), NaCl (200 mM), MgCl_2_ (50 mM), *N*-methylimidazole (50 mM) in HEPES buffer (50 mM, pH 8.0), was incubated at 4 °C for 10 h. A control reaction was run in parallel by replacing Im-p-HH-3′-RNA with unactivated p-HH-3′-RNA. Hammerhead ribozyme cleavage assay: the loop-closing reaction mixture (or control reaction) was diluted 100, 50, or 25 times in water. Then, 1 μL of the diluted reaction mixtures were used to prepare 10 μL of a solution also containing HH-Sub (0.2 μM), NaCl (200 mM), MgCl_2_ (5 mM), and HEPES buffer (50 mM, pH 7.0). Each solution was incubated at 37 °C. (C) Representative PAGE gel electrophoresis for the assembly reaction and the enzymatic assay. Lane 1, the loop-closing ligation after incubating Im-p-HH-3′-RNA (50 μM) and HH-5′- RNA (50 μM) together for 10 h at 4 °C; Lanes 2–13, cleavage of HH-Sub by the reaction mixture after dilution; Lane 14–25, cleavage of the HH-sub by the noncovalently assembled but unligated HH fragments (without loop-closing ligation) after dilution. Yields are average values from duplicate reactions.

In a proof-of-principle experiment, we first targeted the assembly of the well-studied hammerhead ribozyme, the catalytic core of which includes a typical hairpin stem loop.^34^ The full-length hammerhead ribozyme was retrosynthetically disconnected in the loop region, generating a 5′-half fragment (HH-5′-RNA) and a 3′-half fragment (p-HH-3′-RNA) (Figure 4A, B). The HH-5′-RNA strand was labeled with a FAM fluorophore, and the p-HH-3′-RNA strand was converted into the activated phosphorimidazolide form (Im-p-HH-3′-RNA) before mixing with the HH-5′-RNA (each at 50 μM). Under our standard loop-closing ligation conditions, a 12% yield of the full-length hammerhead ribozyme (HH-Full) was observed after 10 h (Figure 4C, Lane 1). We then diluted the loop-closing ligation mixture 250-fold (Figure 4C, Lane 2–5), such that the final concentration of the full-length ribozyme, HH-Full, was about 24 nM, into a solution containing 0.2 μM of the FAM-labeled hammerhead ribozyme substrate (HH-Sub). When we incubated the resulting mixture at 37 °C, the yield of the cleavage product (HH-Pdt) reached 90% (Figure 4C, Lane 2–5) after 4 h.

As a control for the effect of loop-closing ligation, we mixed the HH-5′-RNA with unactivated p-HH-3′-RNA under the same conditions. In this control experiment, we observed only 10% substrate cleavage after a 4 h incubation (Figure 4C, Lane 14–17), which is consistent with previous observations that functional ribozymes can be partially reconstituted by noncovalent association of fragments at high concentrations.^21−24^ When the loop-closing reaction mixture was diluted by 500-fold or 1000-fold (HH-Full was 12 nM or 6 nM), the yields of the cleavage product (HH-Pdt) decreased to 79% and 34% (Figure 4C, Lane 6–13), respectively, after 4 h. However, in these cases, the control experiments with unligated HH fragments (100 nM or 50 nM in concentration) showed no visible cleavage at all (Figure 4C, Lane 18–25).

We also assembled an RNA ligase ribozyme^35^ using a single loop-closing ligation reaction, and efficient ribozyme activity, 35% ligation in 6 h, was observed directly without product purification (Figure S22). These results demonstrate the effectiveness of loop-closing ligation in constructing functional, full-length ribozymes directly in a template-free manner.

## Discussion

The tension between the need for the stable secondary structure in ribozymes and the difficulty posed by the structure for the nonenzymatic (and even ribozyme catalyzed) copying of RNA templates has long been recognized.^21,36^ Our results suggest that loop-closing ligation could have circumvented this problem by enabling the assembly of primordial ribozymes without external templates or splints. This minimizes but does not eliminate the problem of template oligonucleotides inhibiting ribozyme function because the fragments being assembled must still be replicated, and thus, their complementary strands must also reside within the same protocell. We suggest that biased strand synthesis could lead to an excess of one strand over its complement; under conditions favorable to strand annealing, the strand in excess would then exist in solution free of its complementary template. Nonenzymatic RNA template copying is known to be biased in favor of purine rich strands.^37,38^ Furthermore, the loops and internal bulges of aptamers and ribozymes tend to be purine-rich,^39,40^ supporting the hypothesis that asymmetry in strand synthesis coupled with loop-closing ligation would favor the assembly of functional RNAs in primordial protocells. Another possibility arises in our virtual circular genome scenario,^41^ in which the replicating oligonucleotides are fairly short, and under favorable conditions, they are partially base-paired with each other in a large variety of configurations. In this case, inhibition by complementary oligonucleotides might be minimized by their pairing with oligonucleotides from the other strand.

Another potential problem is that 3′-primer extension might out-compete loop-closing ligation. However, if loop-closing involves a 3′-overhang, the 5′-end of the partially complementary oligonucleotide cannot be extended. Even for loop-closing via a 5′-overhang, a possible solution stems from the fact that the rate of primer extension can vary greatly depending on the 3′ nucleotide of the primer and the next two nucleotides of the template. Some of these very slowly extended sequences may correspond to sequences with fast loop-closing kinetics. Additional experiments will be required to explore these possibilities.

The assembly of complex RNA structures by loop-closing ligation also decreases the need for efficient postsynthetic folding of the full-length, single-stranded RNA. Prior to the emergence of RNA chaperones, loop-closing ligation may have provided an internally guided path for the self-assembly of complex structures because the self-assembly process relies upon the prior formation of the correct secondary structure. We suggest that such a self-assembly path may have played key roles in the de novo emergence of structured RNAs at the origin of life.

The loop-closing ligation chemistry that we have described here and our previously described (amino)acyl transfer chemistry both require a close approach of the 5′-end of one strand with the 3′-end of the other strand.^25^ The efficiency of these reactions would be enhanced if the single-stranded overhang sequence was preorganized in a conformation similar to that of the closed loop. The observation that certain overhang sequences result in higher yields of loop-closing ligation (Table 1) strongly suggests that certain sequences are more likely than others to adopt a favorably preorganized conformation. The identification of sequences with optimal preorganization will be important both for understanding the assembly of ribozymes from prebiotically available oligonucleotides, as well as for the design and assembly of ribozymes from an engineering point of view. Our data suggest that loop-closing ligation is more likely to generate 2′-5′-linkages, but 3′-5′-linkages can also be predominant depending on the specific overhang sequence (Figure S20). The observation of efficient ribozyme activity (Figures 3 and S21) suggests that 2′,5′-linkages in loop regions do not have a dramatic impact on these two ribozymes. The relatively low yields of loop-closing ligation in our studies (∼10% observed yield on average) are due in part to the inefficiency of phosphorimidazolide activation, and in part to the competing hydrolysis reaction. A compatible in situ activation chemistry that could maintain sets of oligonucleotides in an activated state^42−44^ might drive these ligation reactions closer to completion and thus favor ribozyme assembly by iterated loop-closing ligations. The efficiency of loop-closing ligation could also be increased by using 3′-amino terminated oligonucleotides, as suggested by our previous work with the splinted ligation of 3′-amino or 2′/3′-aminoacylated nucleotides.^10,17^

The noncovalent assembly of fragments into partly functional ensembles can be seen as an evolutionary precursor to the assembly of full-length ribozymes by loop-closing ligation.^21−24,45^ Notably, it has been demonstrated experimentally that compared to fully random pools of RNA, compact structured pools are superior sources of functional RNAs in in vitro selection experiments.^46,47^ These results strengthen the potential advantage of having direct access to structured, single-stranded RNAs for the emergence of RNA functions. This proposal is in contrast with the conventional view of full-length primary sequences forming first followed by folding. In a pool of short oligonucleotides,^6−8^ loosely structured assemblies of short oligonucleotides with low-level function would have been accessible via dynamic assembly/disassembly of short RNA oligonucleotides at equilibrium. These loosely structured assemblies could have been pulled out-of-equilibrium and trapped as more stable structures by loop-closing ligation, thus enabling the emergence of RNA functions that were more robust than the original noncovalent assembly of fragments. We suggest the straightforward and economical strategy of assembling functional RNA structures by loop-closing ligation is a plausible mechanism for the nonenzymatic emergence of ribozymes before a robust process for the replication of long RNAs was available.

## Supporting Information Available

Loop-closing ligation compared to ligation and acyl-transfer reactions on a nicked duplex and a nicked loop, and nicked duplex construct guided by a template; representative time course of the consumption of Imp-RNA in the loop-closing ligation reaction; scheme for catalysis of loop-closing ligation by *N*-methylimidazole; stacked HPLC traces of loop-closing ligation with UGCCA overhang; stacked HPLC traces of loop-closing ligation with CA overhang; stacked HPLC traces of loop-closing ligation with CCA overhang; stacked HPLC traces of loop-closing ligation with UCA overhang; stacked HPLC traces of loop-closing ligation with UGCCCA overhang; stacked HPLC traces of loop-closing ligation with ACCA overhang; reaction rates of loop-closing ligation depend on the concentration of *N*-methylimidazole (*N*-MeIm); pH dependence of the loop-closing ligation; yields of loop-closing ligation depend on the concentration of MgCl_2_; and yields of loop-closing ligation depend on concentration of NaCl (PDF)
