# Supplementary material for: Template-Free Assembly of Functional RNAs by Loop-Closing Ligation
Source: J Am Chem Soc. 2022 Jul 26;144(30):13920–7. doi: 10.1021/jacs.2c05601 (PMC9354263; doi:10.1021/jacs.2c05601)
Supplement: Supplementary file 1 — ja2c05601_si_001.pdf [file ja2c05601_si_001.pdf]

Supporting information for

Template-free assembly of functional RNAs by loop-closing ligation

Long-Fei Wu[†,‡,§,¶], Ziwei Liu[†], Samuel J. Roberts[†], Meng Su[†], Jack W. Szostak*[,‡,§,¶] and John D. Sutherland*[,†]

[†]MRC Laboratory of Molecular Biology, Francis Crick Avenue, Cambridge Biomedical Campus, Cambridge, CB2 0QH, United Kingdom.

[‡]Howard Hughes Medical Institute, Department of Molecular Biology, and Center for Computational and Integrative Biology, Massachusetts General Hospital, Boston, Massachusetts 02114, United States.

[§]Department of Genetics, Harvard Medical School, Boston, Massachusetts 02115, United States.

[¶]Department of Chemistry and Chemical Biology, Harvard University, Cambridge, Massachusetts 02138, United States.

*Correspondence to: szostak@molbio.mgh.harvard.edu, johns@mrc-lmb.cam.ac.uk.

Table of contents

1. Materials and General………………………………………………………… 2
2. Methods…………………………………………………………………………… 3
3. Supplementary Figures ..……………………………………………………… 8
4. Supplementary Tables…………………………………………………………… 30

Materials and General

Reagents and solvents were obtained from *Acros Organics*, *Alfa Aesar*, *Santa Cruz Biotechnology*, *Sigma-Aldrich*, *SYNTHON Chemicals GmbH & Co. KG* and *VWR International*, and were used without further purification unless otherwise stated. For solid phase RNA synthesis, primer Support 5G for A, G, C, U or 2′-dA (with loading ~300 µmol/g) was purchased from GE Healthcare. 3′-dA-CPG (with loading 50 µmol/g, item number 20-2004-01) was purchased from Glen Research. Phosphoramidites for RNA synthesis were purchased from Sigma-Aldrich or Link Technologies. RNA oligomers used in this study were synthesized using an ÄKTA™ oligopilot™ plus 10 (*GE Healthcare*) on a 5 to 50 µmol scale or were synthesised using an Expedite 8909 on 1 µmol scale. A *MettlerToledo* SevenEasy pH Meter S20 combined with a *ThermoFisher Scientific* Orion 8103BN Ross semi-micro pH electrode was used to measure and adjust the pH to the desired value. High-Pressure Liquid Chromatography (HPLC) was run on Dionex Ultimate 3000 (Thermo Scientific) using an Atlantis™ T3, 5 µm, 4.6 x 250 mm column or an Atlantis™ T3, 3 µm, 4.6 x 150 mm column. Polyacrylamide gel electrophoresis: 20 % polyacrylamide, 8 M urea gels (0.75 mm thick, 20 cm long) were run at 18 W in 1X TBE buffer (89 mM Tris-borate and 2 mM EDTA, pH 8.3) for 1 hours. FAM-labeled RNA oligomers were detected and imaged with an Amersham RGB Biomolecular Imager (GE Healthcare Life Science, Marlborough, MA) and quantified with the ImageQuant™ software package (GE Healthcare Life Science, Marlborough, MA). To image unlabeled RNA oligos, the RNA gel was stained using SYBR Gold Nucleic Acid Gel Stain (Invitrogen). Oligonucleotide concentrations were determined by UV absorbance at 260 nm using a NanoDrop® ND-1000 spectrophotometer.

Methods

Solid phase synthesis of RNA oligomers

After automated synthesis, RNAs were cleaved from the solid support by treating with 3 mL (for 5-50 μmol scale synthesis, if 1 μmol scale then 1.2 mL of mixture was used) of a 1:1 mixture of 28% wt NH_3/H_2O solution and 33% wt CH_3NH_2/EtOH solution at 55 °C for 30 minutes in a tube with a sealed cap. The solid was removed by filtration and washed with 50 % EtOH/H_2O. The solution and washings were combined and evaporated to dryness under reduced pressure. Silyl protecting groups were then removed by treating the residues with 3 mL (for 5-50 μmol scale synthesis, if 1 μmol scale then 0.25 mL of mixture was used) of 1:1 mixture of triethylamine trihydrofluoride and DMSO at 55°C for 90 minutes in a tube with a sealed cap. After brief cooling at -32 °C, 30 mL of cold 50 mM $NaClO_4$ in acetone was added to the solution to precipitate the RNA product. The resulting mixture was centrifuged and the pellet of RNA was re-dissolved in 10 mL of water and passed through a *Waters* Sep-Pack C18 Cartridge, 5 g sorbent (Cartridge was pre-washed with 20 mL of MeOH then 100 mL of water before sample loading, then washed with 100 mL of H_2O, 20 mL of 10 % MeOH/H_2O, 25 mL of 20 % MeOH/H_2O, 25 mL of 50 % MeOH/H_2O and 20 mL of MeOH sequentially). Eluates containing RNA were combined and lyophilized. The resulting RNA was stored as a solid or dissolved in neutral pH solution at -32 °C for future usage.

General procedure for chemical synthesis of Im-p-RNA

An aqueous reaction mixture (300 μL), containing the 5′-phosphoryl RNA (0.1 to 2 mM) and imidazole (80 mM), was titrated to pH 7. Then EDC (4.8 mg, final concentration 80 mM) was added, and the reaction mixture was incubated at room temperature. After 2 hours, 10 mL of cold 50 mM of $NaClO_4$ in acetone were added to the reaction mixture to precipitate the RNA oligomers. The resulting cloudy mixture was shaken intensively and then placed in a -32 °C freezer for half an hour. The white pellet of product obtained by centrifugation was washed twice with 2 mL of cold 50 mM of $NaClO_4$ in acetone, then dried in a desiccator under vacuum for half an hour.

Finally, the white pellet was dissolved in 60-300 μL (making combined concentration of RNA above 0.5 mM, including both 5′-p-RNA and 5′-Im-p-RNA) of 20 mM HEPES buffer with pH 8.0 and stored at -32 °C for future usage without purification. The combined concentration of 5′-Im-p-RNA and 5′-p-RNA was measured by UV absorbance at 260 nm using a NanoDrop. The yields of Im-p-RNA based on initial 5′-p-RNA ranged from 40 % to 80 % as measured by HPLC analysis.

Standard procedure for loop-closing ligation (Table 1, Figure S4-S19)

A 50 μL reaction mixture containing Im-p-AGCGA (50 μM total, including both Im-p-AGCGA and p-AGCGA), phosphate acceptor RNA (50 μM, 5′-UCGCUUGCCA-3′), *N*-methylimidazole (MeIm, 50 mM, added last to initiate the catalytic reaction), NaCl (200 mM), $MgCl_2$ (50 mM), cytosine (100 μM, internal reference for HPLC analysis), in HEPES buffer (50 mM, pH 8.0) was incubated at 20 °C. Aliquots (8 μL) were taken at specific time points and injected directly into an HPLC for analysis with 260 nm UV detection (Atlantis^{TM} T3, 5 μm, 4.6 x 250 mm column; flow rate 1 mL/min; LC solvents: A, 25 mM triethylammonium acetate, pH 7.5 in water and B, acetonitrile. Column compartment temperature was 25 °C).

The observed yield of loop-closing ligation was calculated by comparing the above reaction to a parallel reaction run at pH 5.2 (MES buffer, 50 mM). At pH 5.2, Im-p-AGCGA hydrolysed exclusively to p-AGCGA without loop-closing ligation. The integrated areas of p-AGCCA peaks of reactions at pH 5.2 and at condition of interest were used to calculate the observed yields. Reactions with altered pH, temperature, concentration of *N*-MeIm, NaCl, $MgCl_2$ were analysed similarly.

Corrected yields of the loop-closing ligation: Loop-closing ligation yields were corrected on the basis of the measured amounts of Im-p-AGCGA and 5′-p- AGCGA in the starting samples. The corrected yields represent the partition of Im-p-AGCGA into loop-closing ligation product vs. hydrolysis under a certain condition. The corrected yield does not change as the percentage of Im-p-AGCGA varies between synthetic batches, but the observed yields do.

Regioselectivity (2′-5′- versus 3′-5′-phosphodiester) of the loop-closing ligation in the model reactions (Figure S20)

1 μL of the above loop-closing ligation reaction was quenched with 40 μL of stop buffer (6 M urea in TEB buffer with 100 mM EDTA, pH 8.0). An all- 3′-5′-linkage authentic standard and an authentic standard with one 2′-5′-linkage at the loop-closing position were prepared in stop buffer at 0.5 μM. 2 μL of the quenched and standard solutions, were analysed by PAGE. The regioselectivity of the newly formed phosphodiester bond was assigned by comparing the reactions to the standards by imaging after SYBR Gold Nucleic Acid Gel Staining.

Assembly of a tRNA minihelix structure by loop-closing ligation and nicked duplex ligation (Figure 3 and Figure S21)

To a 10 μL reaction mixture containing 5′-FAM-AUUAGGAGAUG-3′ (RNA-1, 25 μM), 5′-Im-p-GAGGGUUUGAGA-3′ (Im-p-RNA-2, 25 μM in total, including 5′-Im-p-GAGGGUUUGAGA-3′ and 5′-p-GAGGGUUUGAGA-3′), 5′-Im-p-CCCUUCAUCUCCACCA-3′ (Im-p-RNA-3, 25 μM in total, including 5′-Im-p-CCCUUCAUCUCCACCA-3′ and 5'-p-CCCUUCAUCUCCACCA-3'), NaCl (200 mM), $MgCl_2$ (50 mM) in HEPES buffer (50 mM, pH 8.0) was added *N*-methylimidazole (MeIm, 50 mM), followed by incubation at 25 °C. Aliquots (0.5 μL) were taken at specific time points and quenched in 25 μL of stop buffer (6 M urea in TBE buffer with 100 mM EDTA, pH 8.0). 2 μL of the quenched solution was analysed by PAGE. Observed yields were quantified according to the relative amounts of FAM-labelled oligomers by gel imaging. Products without FAM-labelling was visualised, but not quantified, after SYBR Gold Nucleic Acid Gel Staining.

Assembly of the full-length hammerhead ribozyme by loop-closing ligation and subsequent enzymatic assay (Figure 4)

A 10 μL reaction mixture containing 5′-FAM-ACCUGUCUGAUGAGCAAG-3′ (HH-5'-RNA, 50 μM), 5′-Imp-UUAUCUUGCGAAACCGU-3′ (Im-p-HH-3′-RNA, 50 μM, including 5′-Imp-UUAUCUUGCGAAACCGU-3′ and 5′-p-UUAUCUUGCGAAA-

CCGU-3′), *N*-methylimidazole (MeIm, 50 mM, added last to initiate the catalytic reaction), NaCl (200 mM), $MgCl_2$ (50 mM) in HEPES buffer (50 mM, pH 8.0) was incubated at 4 °C. A control reaction was run in parallel by replacing Im-p-HH-3′-RNA with unactivated p-HH-3′-RNA (5′-p-UUAUCUUGCGAAACCGU-3′, 50 µM). After 10 hours, 0.5 µL of the reaction solution was diluted in 20 µL of water, then 1 µL of the diluted solution was quenched in 9 uL of stop solution (6 M urea in TEB buffer with 100 mM EDTA, pH 8.0). 2 µL of the quenched solution was analysed by PAGE. Observed yields of the full-length hammerhead ribozyme (HH-Full) were quantified according to the relative amounts of FAM-labelled oligomers by gel imaging.

Hammerhead ribozyme assay: The loop-closing reaction mixture was diluted 100, 50, or 25 times, respectively, in water, and dilutions of the control reaction without loop-closing ligation were also prepared. Then, 1 µL of the previously diluted reaction mixtures were used to prepare 10 µL of a solution also containing 5′-FAM-AAACGGUCACAGGU-3′ (HH-Sub, 0.2 µM), NaCl (200 mM), $MgCl_2$ (5 mM) and HEPES buffer (50 mM, pH 7.0). Each solution was incubated at 37 °C, and 2 µL of reaction mixture was quenched by adding to 6 µL of stop solution (6 M urea in TEB buffer with 100 mM EDTA, pH 8.0) at t = 1, 2, 3 and 4 hours, respectively. 2 µL of the quenched solution was analysed by PAGE. Yields of cleavage of HH-Sub (5′-FAM-AAACGGUCACAGGU-3′) to the 8-nt product (HH-Pdt, 5′-FAM-AAACGGUC>p) were quantified by gel imaging.

Assembly of the full-length Joyce ligase ribozyme by loop-closing ligation and subsequent enzymatic assay (Figure S22)

A 10 µL reaction mixture containing 5′-FAM-UAAAGUUGUUAUCACU-CGUAGUUCCA-3′, (Lig-5′-RNA, 50 µM), 5′-Imp-CUACGUUAUGGAUGGGUU-GAAGUAU-3′, (Im-p-Lig-3′-RNA, 50 µM, including 5′-Imp-CUACGUUAUGGA-UGGGUUGAAGUAU-3′ and 5′-p-CUACGUUAUGGAUGGGUUGAAGUAU-3′), *N*-methylimidazole (*N*-MeIm, 50 mM, added last to initiate the catalytic reaction), NaCl (200 mM), $MgCl_2$ (50 mM) in HEPES buffer (50 mM, pH 8.0) was incubated at 25 °C. A control reaction was run in parallel by replacing Im-p-Lig-3′-RNA with unactivated

p-Lig-3′-RNA (5′-p-CUACGUUAUGGAUGGGUUGAAGUAU-3′, 50 μM). After 10 hours, 0.5 μL of the reaction solution was diluted in 20 μL of water, then 1 μL of the diluted solution was quenched in 9 uL of stop solution (6 M urea in TEB buffer with 100 mM EDTA, pH 8.0). 2 μL of the quenched solution was analysed by PAGE. Yields of the full-length ligase ribozyme (Lig-Full) were quantified by fluorescence gel imaging.

Ligase ribozyme assay: A 10 μL enzymatic reaction mixture containing ppp-GAGACCGCAACUUA (Lig-Sub, 4 μM), NaCl (200 mM), $MgCl_2$ (50 mM), HEPES buffer (50 mM, pH 8.0) and ~ 0.8 μM of Lig-Full was prepared. The Lig-Full solution (2 μL) was added last. The same procedure was applied to the control reaction without loop-closing ligation. The resulting solutions were incubated at 48 °C for 6 hours, then 1 μL of each reaction mixture was quenched by addition to 9 μL of stop solution (6 M urea in TBE buffer with 100 mM EDTA, pH 8.0). 2 μL of the quenched solution was analysed by PAGE. The yield of the enzymatic ligation product (Lig-Ptd) was quantified by gel imaging. Products without FAM-label were visualised, but not quantified, by SYBR Gold Nucleic Acid Gel Staining

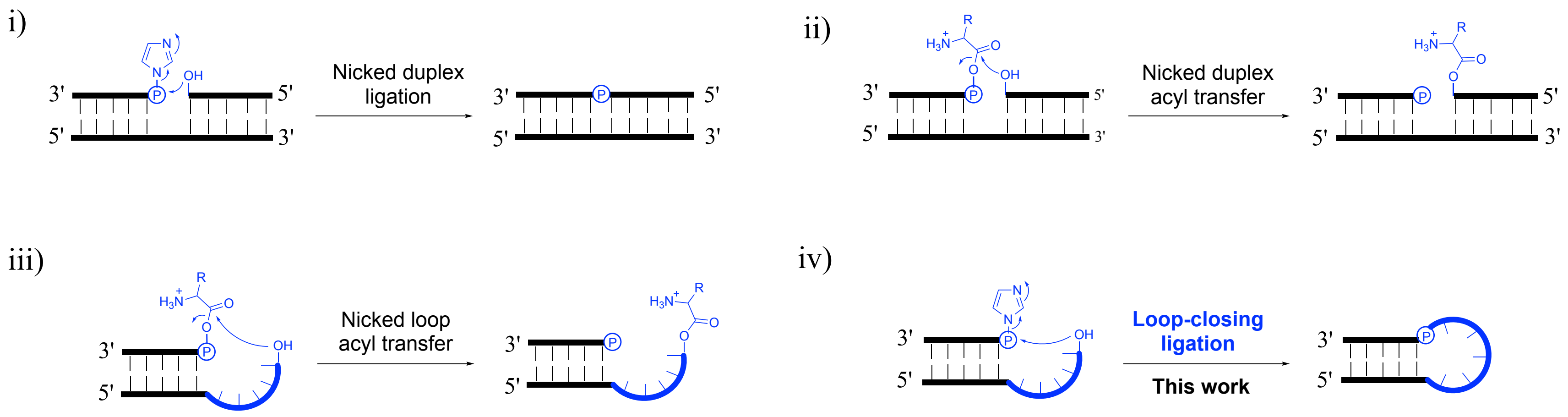

Figure S1. Loop-closing ligation compared to ligation and acyl-transfer reactions on a nicked duplex and a nicked loop, and nicked duplex construct guided by a template: i) The proximity of 5′- and 3′-ends in a nicked duplex facilitates ligation when the 5′-phosphate was activation, and also, ii) facilitates acyl transfer chemistry from a 5′-mixed anhydride. **Nicked loop construct without external template**: iii) The proximity of 5′- and 3′-ends in a nicked loop facilitates acyl transfer chemistry from a 5′-mixed anhydride, which suggested that iv) the same proximity might facilitate ligation chemistry in a nicked loop, for example using 5′-phosphorimidazolide activation.

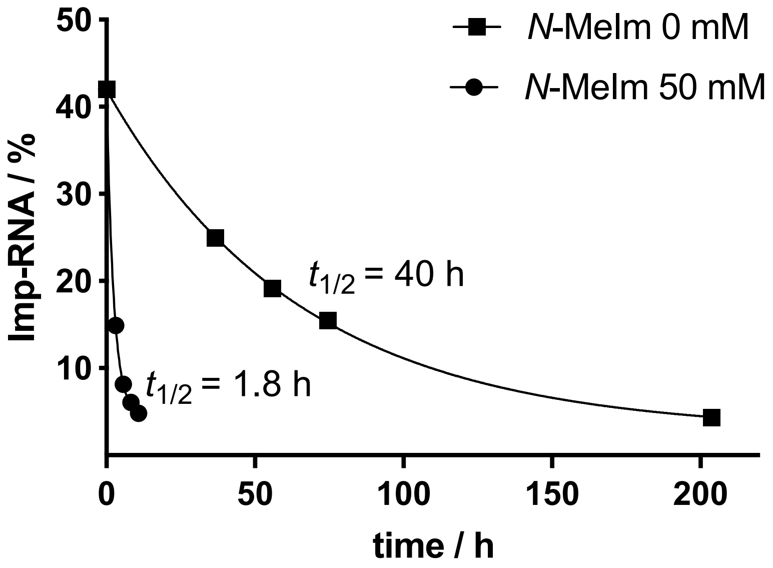

Figure S2. Representative time course of the consumption of Imp-RNA in the loop-closing ligation reaction. Reaction condition: 50 μM each of the phosphate donor and phosphate acceptor RNA strands, *N*-MeIm (0 mM or 50 mM), $MgCl_2$, 50 mM, NaCl 200 mM, HEPES 50 mM, pH 8.0 at 20 °C. Half-lives included in the figure are average values of duplicates.

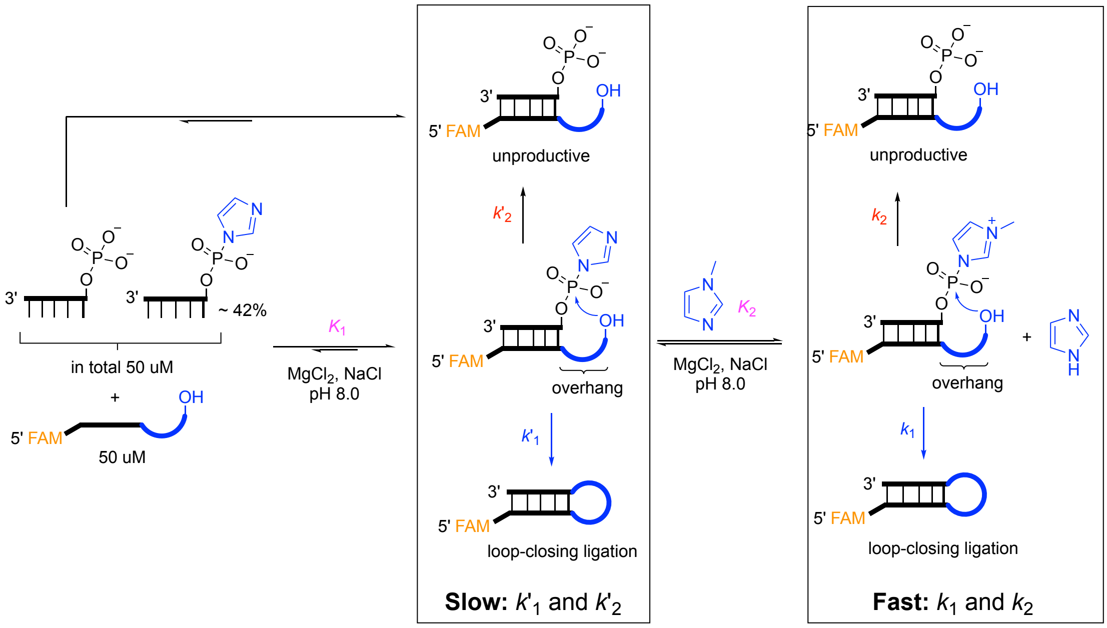

Figure S3. Scheme for catalysis of loop-closing ligation by *N*-methylimidazole. The equilibrium between Im-p-AGCGA + *N*-MeIm and *N*-MeIm-p-AGCGA + Im could also happen off-duplex, and all the other possible association and dissociation equilibria of RNA strands are omitted for simplicity. The Imp-RNA percentage, [Imp-RNA]/([Imp-RNA] + [p-RNA]), from the starting material (including Imp-RNA and p-RNA) varies from batch to batch. Standard reaction condition: 50 µM each of the phosphate donor and phosphate acceptor RNA strands, *N*-MeIm 50 mM, $MgCl_2$, 50 mM, NaCl 200 mM, HEPES 50 mM, pH 8.0 at 20 °C. Im, imidazole; *N*-MeIm, *N*-methylimidazole. The overhang sequence and imidazole leaving groups are highlighted in blue.

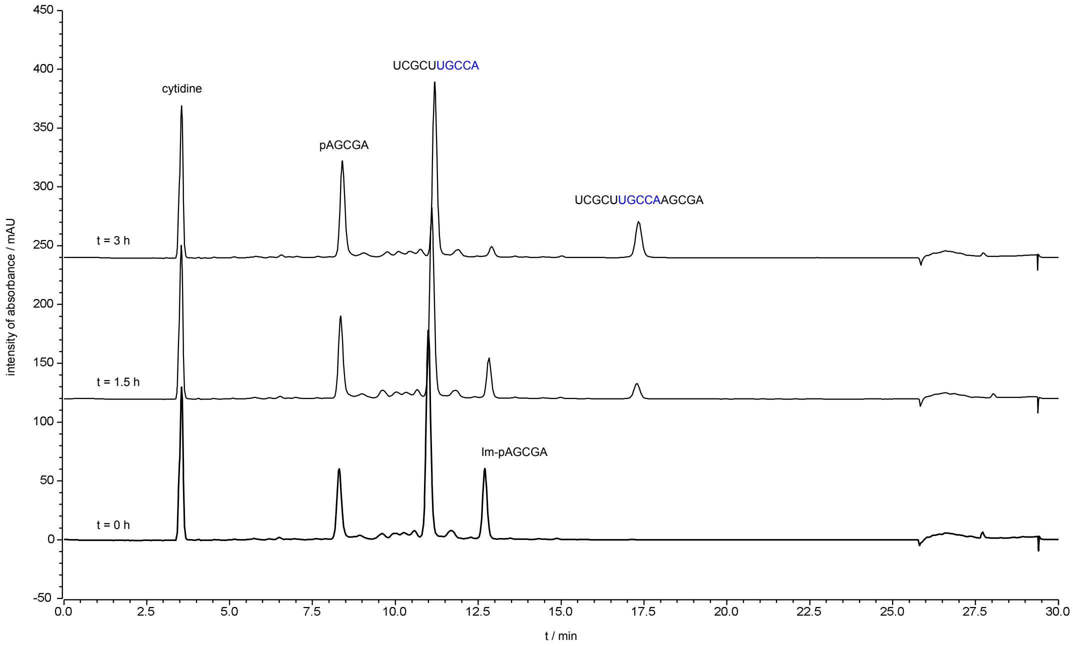

Figure S4. Stacked HPLC traces of loop-closing ligation with UGCCA overhang. Loop duplex sequence:

`3'AGCGAp-Im`

`5'UCGCUUGCCA`

Loop-closing ligation was monitored by HPLC with 260 nm UV detection. The solution was incubated at 20 °C and aliquots of 8 μL were injected into an HPLC at different time points. Peaks for the phosphate donor, phosphate acceptor strands and the product of loop-closing ligation are indicated. Conditions: 50 μL of reaction mixture, containing the phosphate donor strand (including Im-p-AGCGA and p-AGCGA, in total 50 μM), the phosphate acceptor strand (5′-UCGCUUGCCA-3′, 50 μM), cytidine (internal standard, 200 μM), NaCl (200 mM), $MgCl_2$ (50 mM), *N*-MeIm (50 mM) and HEPES (50 mM, pH 8), was incubated at 20 °C.

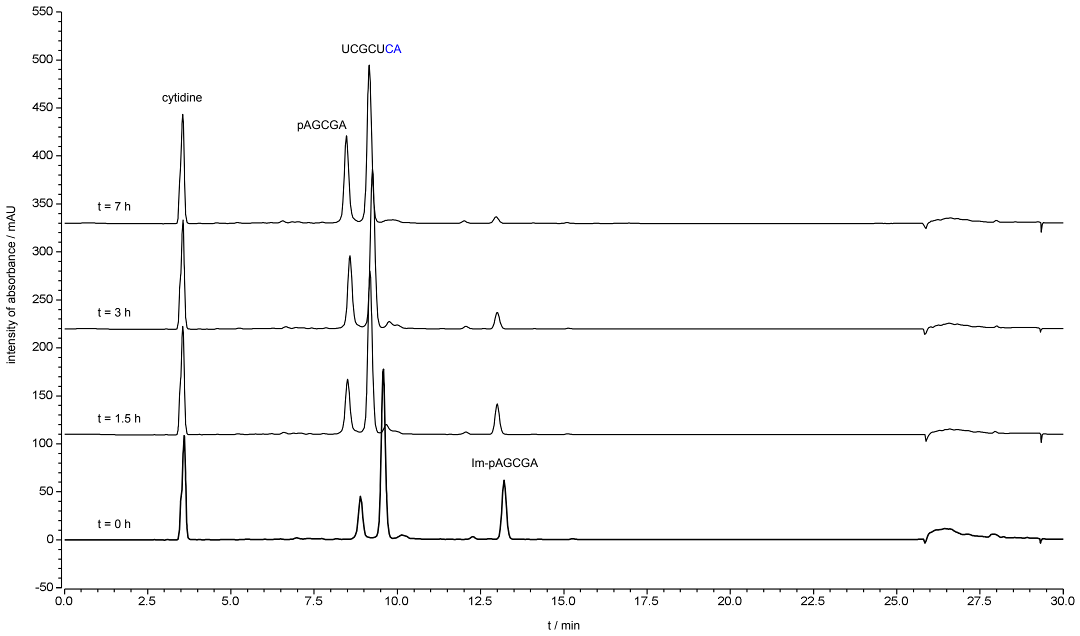

Figure S5. Stacked HPLC traces of loop-closing ligation with CA overhang. Loop duplex sequence:

3′AGCGAp-Im

5′UCGCUCA

Loop-closing ligation was monitored by HPLC with 260 nm UV detection. The solution was incubated at 20 °C and aliquots of 8 µL were injected into an HPLC at different time points. Peaks for the phosphate donor, phosphate acceptor strands and the product of loop-closing ligation are indicated. Conditions: 50 µL of reaction mixture, containing the phosphate donor strand (including Im-p-AGCGA and p-AGCGA, in total 50 µM), the phosphate acceptor strand (5′-UCGCUCA-3′, 50 µM), cytidine (internal standard, 200 µM), NaCl (200 mM), $MgCl_2$ (50 mM), *N*-MeIm (50 mM) and HEPES (50 mM, pH 8), was incubated at 20 °C.

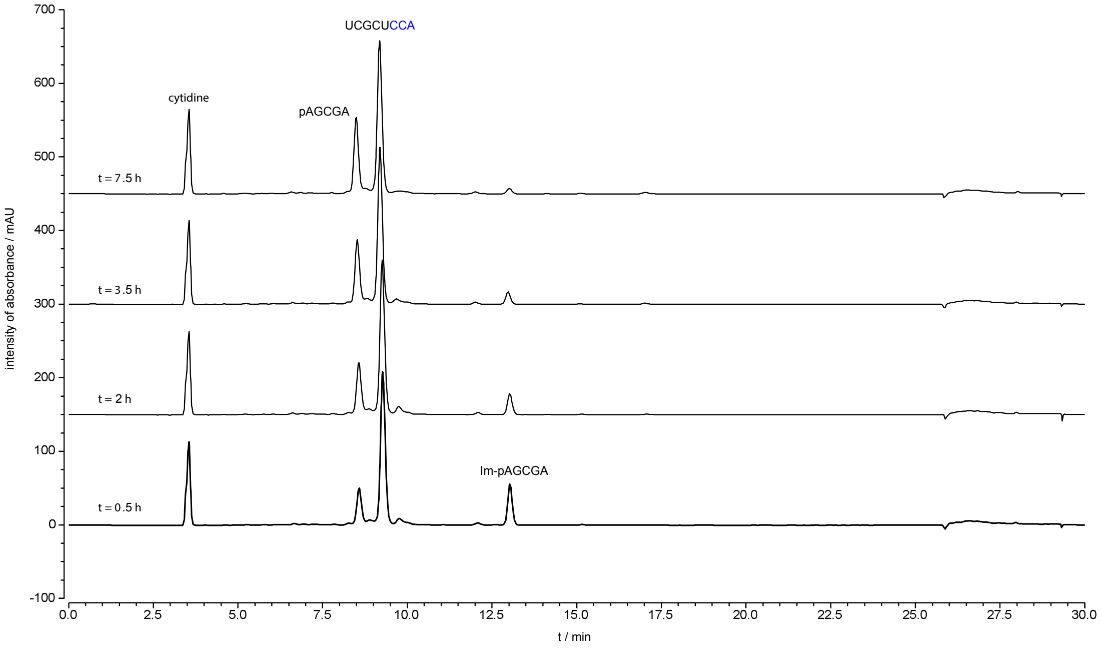

Figure S6. Stacked HPLC traces of loop-closing ligation with CCA overhang. Loop duplex sequence:

`3'AGCGAp-Im`

`5'UCGCUCCA`

Loop-closing ligation was monitored by HPLC with 260 nm UV detection. The solution was incubated at 20 °C and aliquots of 8 µL were injected into an HPLC at different time points. Peaks for the phosphate donor, phosphate acceptor strands and the product of loop-closing ligation are indicated. Conditions: 50 µL of reaction mixture, containing the phosphate donor strand (including Im-p-AGCGA and p-AGCGA, in total 50 µM), the phosphate acceptor strand (5′-UCGCUCCA-3′, 50 µM), cytidine (internal standard, 200 µM), NaCl (200 mM), $MgCl_2$ (50 mM), *N*-MeIm (50 mM) and HEPES (50 mM, pH 8), was incubated at 20 °C.

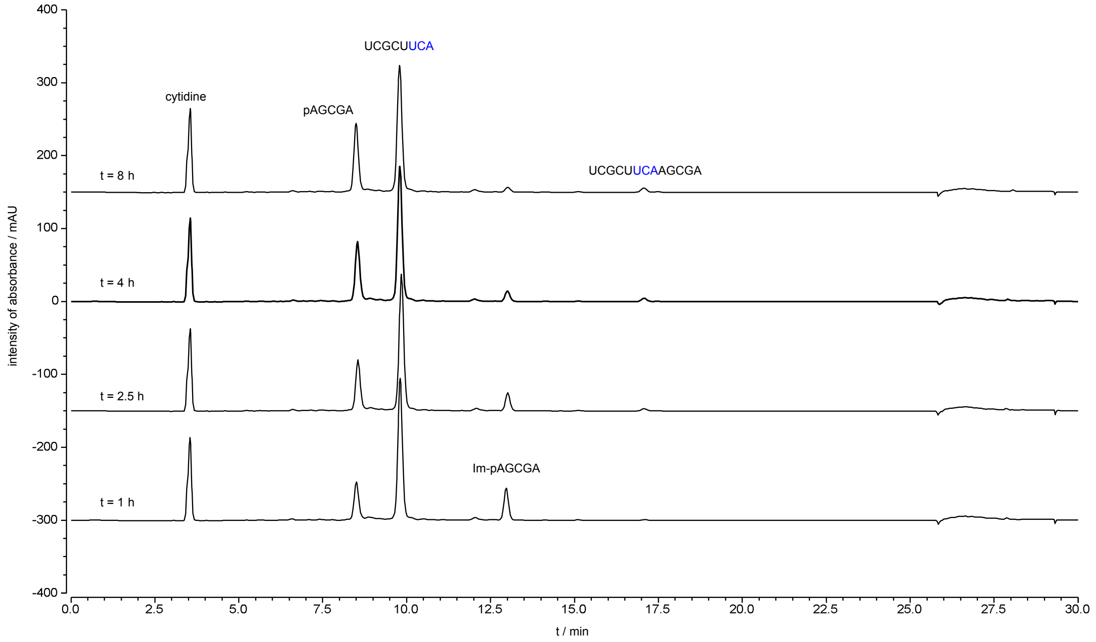

Figure S7. Stacked HPLC traces of loop-closing ligation with UCA overhang. Loop duplex sequence:

3'AGCGAp-Im

5'UCGCUUCA

Loop-closing ligation was monitored by HPLC with 260 nm UV detection. The solution was incubated at 20 °C and aliquots of 8 µL were injected into an HPLC at different time points. Peaks for the phosphate donor, phosphate acceptor strands and the product of loop-closing ligation are indicated. Conditions: 50 µL of reaction mixture, containing the phosphate donor strand (including Im-p-AGCGA and p-AGCGA, in total 50 µM), the phosphate acceptor strand (5′-UCGCUUCA-3′, 50 µM), cytidine (internal standard, 200 µM), NaCl (200 mM), $MgCl_2$ (50 mM), *N*-MeIm (50 mM) and HEPES (50 mM, pH 8), was incubated at 20 °C.

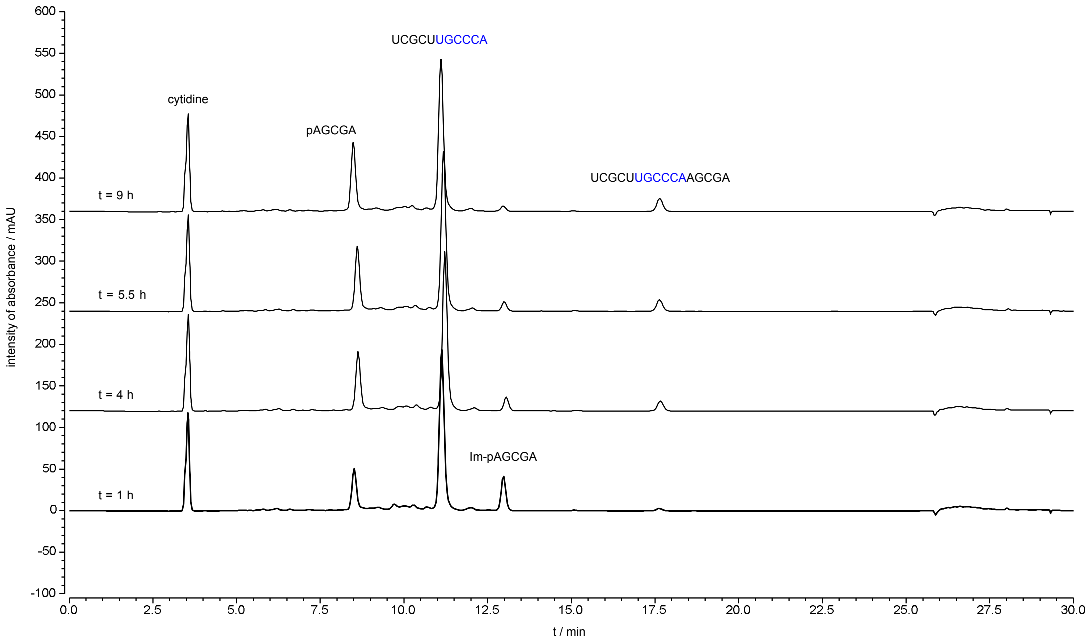

Figure S8. Stacked HPLC traces of loop-closing ligation with UGCCCA overhang. Loop duplex sequence:

3′AGCGAp-Im

5′UCGCUUGCCCA

Loop-closing ligation was monitored by HPLC with 260 nm UV detection. The solution was incubated at 20 °C and aliquots of 8 µL were injected into an HPLC at different time points. Peaks for the phosphate donor, phosphate acceptor strands and the product of loop-closing ligation are indicated. Conditions: 50 µL of reaction mixture, containing the phosphate donor strand (including Im-p-AGCGA and p-AGCGA, in total 50 µM), the phosphate acceptor strand (5′-UCGCUUGCCCA-3′, 50 µM), cytidine (internal standard, 200 µM), NaCl (200 mM), $MgCl_2$ (50 mM), *N*-MeIm (50 mM) and HEPES (50 mM, pH 8), was incubated at 20 °C.

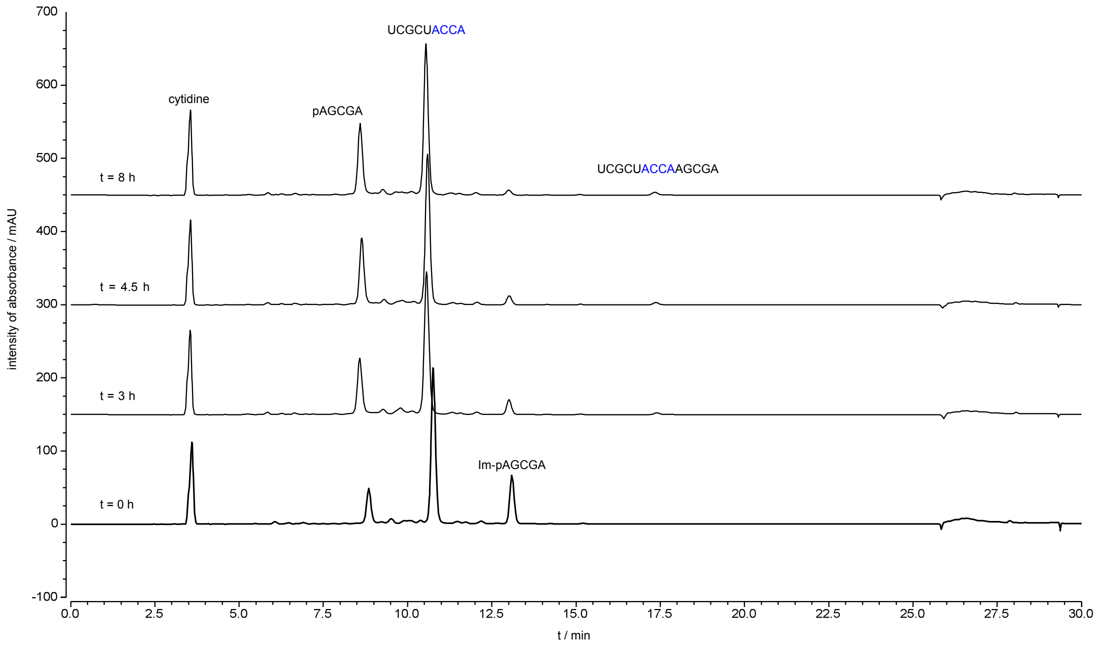

Figure S9. Stacked HPLC traces of loop-closing ligation with ACCA overhang. Loop duplex sequence:

`3'AGCGAp-Im`

`5'UCGCUACCA`

Loop-closing ligation was monitored by HPLC with 260 nm UV detection. The solution was incubated at 20 °C and aliquots of 8 μL were injected into an HPLC at different time points. Peaks for the phosphate donor, phosphate acceptor strands and the product of loop-closing ligation are indicated. Conditions: 50 μL of reaction mixture, containing the phosphate donor strand (including Im-p-AGCGA and p-AGCGA, in total 50 μM), the phosphate acceptor strand (5′-UCGCUACCA-3′, 50 μM), cytidine (internal standard, 200 μM), NaCl (200 mM), $MgCl_2$ (50 mM), *N*-MeIm (50 mM) and HEPES (50 mM, pH 8), was incubated at 20 °C.

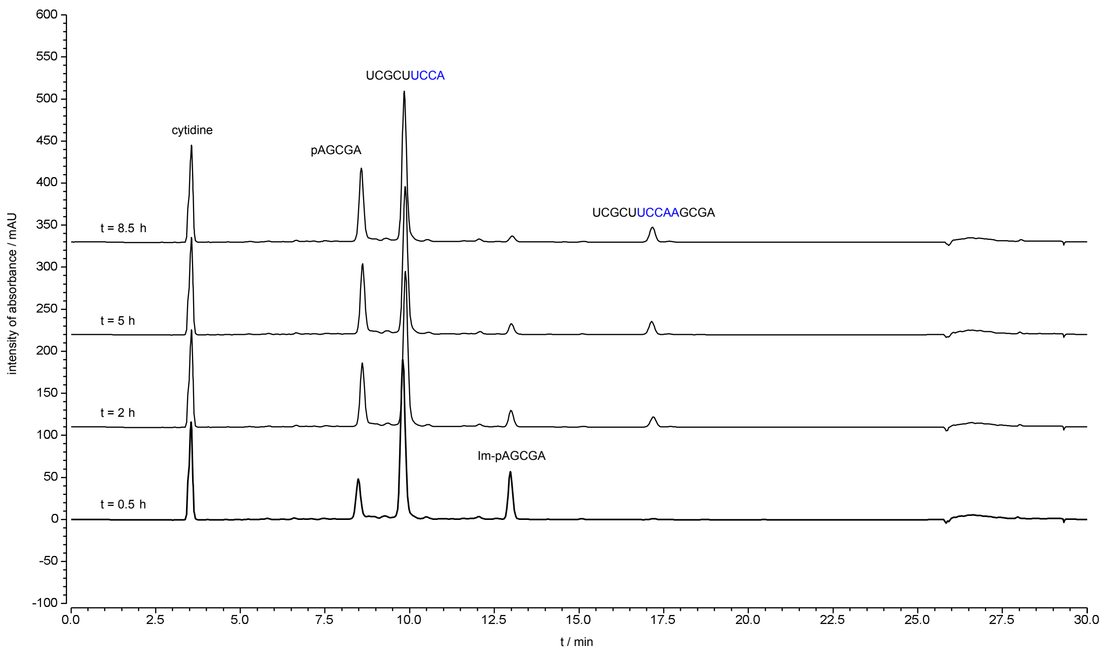

Figure S10. Stacked HPLC traces of loop-closing ligation with UCCA overhang.

Loop duplex sequence:

`3'AGCGAp-Im`

`5'UCGCUUCCA`

Loop-closing ligation was monitored by HPLC with 260 nm UV detection. The solution was incubated at 20 °C and aliquots of 8 μL were injected into an HPLC at different time points. Peaks for the phosphate donor, phosphate acceptor strands and the product of loop-closing ligation are indicated. Conditions: 50 μL of reaction mixture, containing the phosphate donor strand (including Im-p-AGCGA and p-AGCGA, in total 50 μM), the phosphate acceptor strand (5′-UCGCUUCCA-3′, 50 μM), cytidine (internal standard, 200 μM), NaCl (200 mM), $MgCl_2$ (50 mM), *N*-MeIm (50 mM) and HEPES (50 mM, pH 8), was incubated at 20 °C.

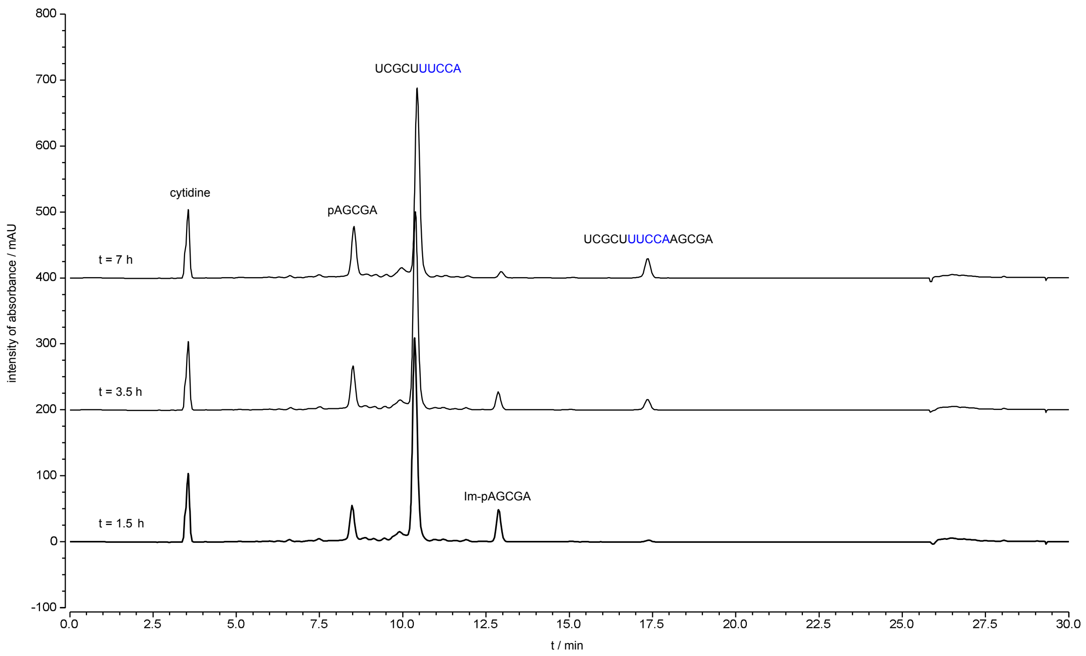

Figure S11. Stacked HPLC traces of loop-closing ligation with UUCCA overhang.

Loop duplex sequence:

3′AGCGAp-Im

5′UCGCUUUCCA

Loop-closing ligation was monitored by HPLC with 260 nm UV detection. The solution was incubated at 20 °C and aliquots of 8 µL were injected into an HPLC at different time points. Peaks for the phosphate donor, phosphate acceptor strands and the product of loop-closing ligation are indicated. Conditions: 50 µL of reaction mixture, containing the phosphate donor strand (including Im-p-AGCGA and p-AGCGA, in total 50 µM), the phosphate acceptor strand (5′-UCGCUUUCCA-3′, 50 µM), cytidine (internal standard, 200 µM), NaCl (200 mM), $MgCl_2$ (50 mM), *N*-MeIm (50 mM) and HEPES (50 mM, pH 8), was incubated at 20 °C.

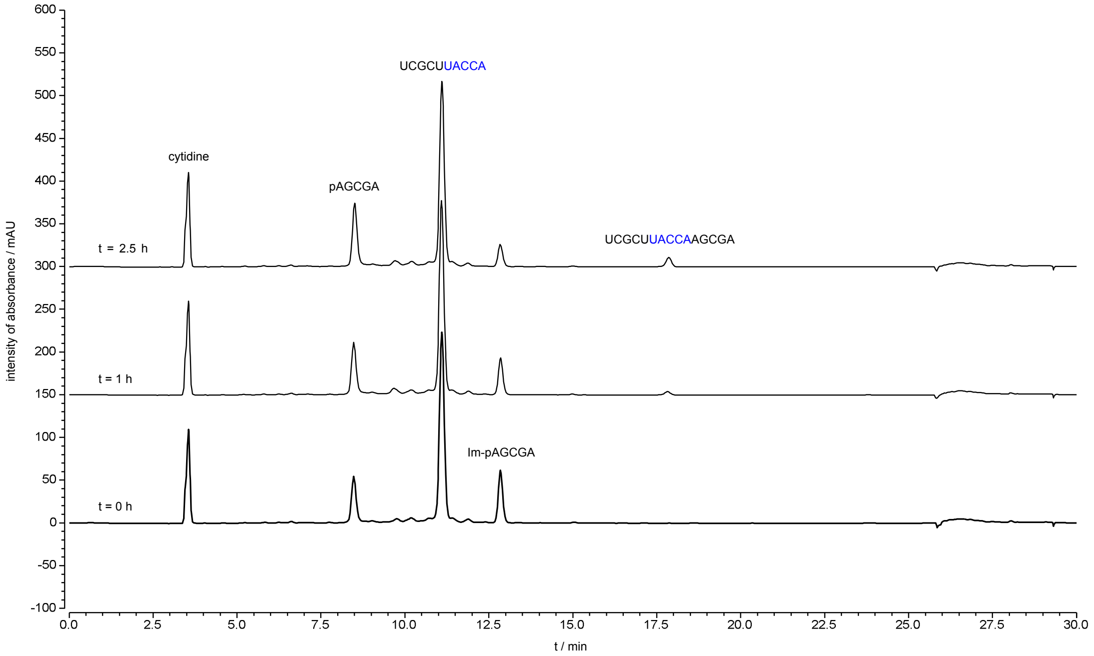

Figure S12. Stacked HPLC traces of loop-closing ligation with UACCA overhang.

Loop duplex sequence:

`3'AGCGAp-Im`

`5'UCGCUUACCA`

Loop-closing ligation was monitored by HPLC with 260 nm UV detection. The solution was incubated at 20 °C and aliquots of 8 µL were injected into an HPLC at different time points. Peaks for the phosphate donor, phosphate acceptor strands and the product of loop-closing ligation are indicated. Conditions: 50 µL of reaction mixture, containing the phosphate donor strand (including Im-p-AGCGA and p-AGCGA, in total 50 µM), the phosphate acceptor strand (5′-UCGCUUACCA-3′, 50 µM), cytidine (internal standard, 200 µM), NaCl (200 mM), $MgCl_2$ (50 mM), *N*-MeIm (50 mM) and HEPES (50 mM, pH 8), was incubated at 20 °C.

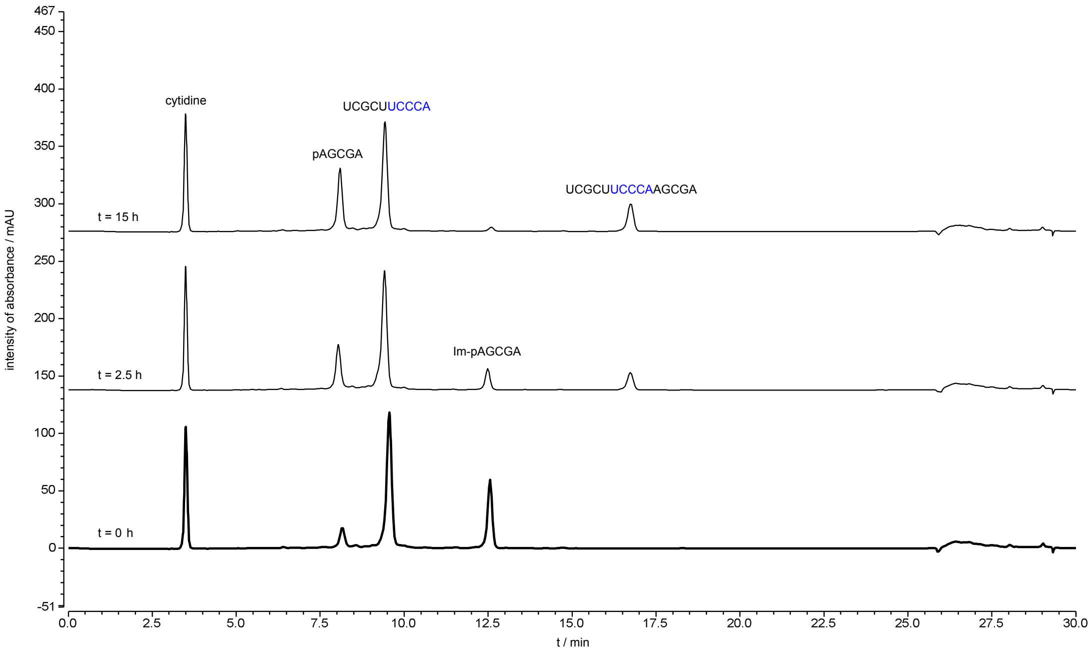

Figure S13. Stacked HPLC traces of loop-closing ligation with UCCCA overhang.

Loop duplex sequence:

```
3'AGCGAp-Im
5'UCGCUUCCCA
```

Loop-closing ligation was monitored by HPLC with 260 nm UV detection. The solution was incubated at 20 °C and aliquots of 8 µL were injected into an HPLC at different time points. Peaks for the phosphate donor, phosphate acceptor strands and the product of loop-closing ligation are indicated. Conditions: 50 µL of reaction mixture, containing the phosphate donor strand (including Im-p-AGCGA and p-AGCGA, in total 50 µM), the phosphate acceptor strand (5′-UCGCUUCCCA-3′, 50 µM), cytidine (internal standard, 200 µM), NaCl (200 mM), $MgCl_2$ (50 mM), *N*-MeIm (50 mM) and HEPES (50 mM, pH 8), was incubated at 20 °C.

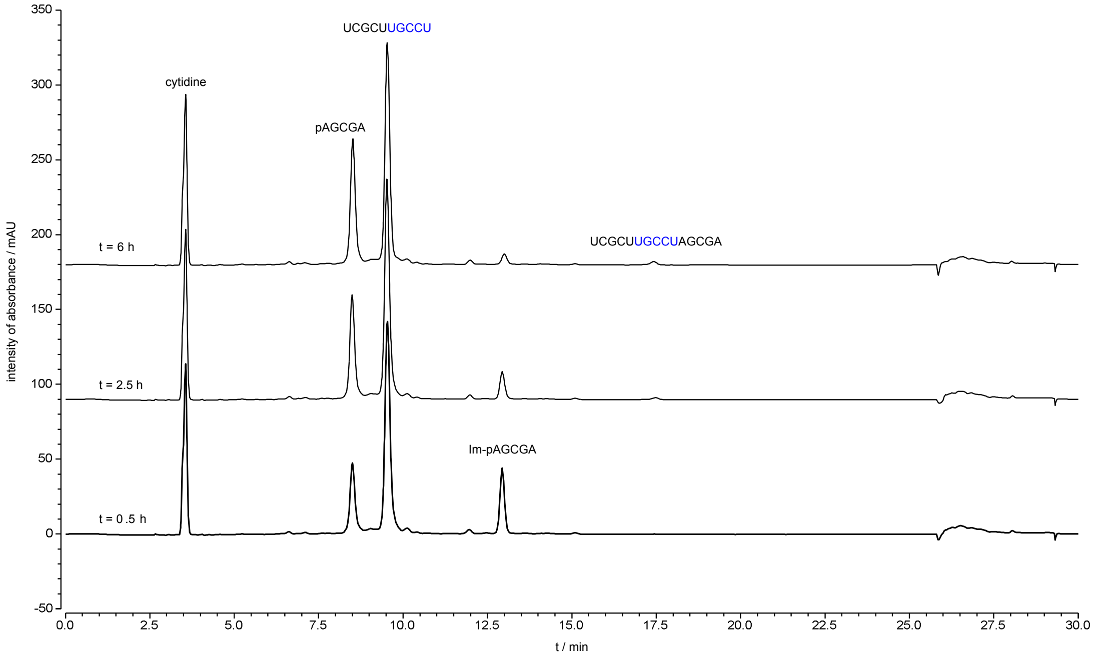

Figure S14. Stacked HPLC traces of loop-closing ligation with UGCCU overhang.

Loop duplex sequence:

```
3'AGCGAp-Im
5'UCGCUUGCCU
```

Loop-closing ligation was monitored by HPLC with 260 nm UV detection. The solution was incubated at 20 °C and aliquots of 8 μL were injected into an HPLC at different time points. Peaks for the phosphate donor, phosphate acceptor strands and the product of loop-closing ligation are indicated. Conditions: 50 μL of reaction mixture, containing the phosphate donor strand (including Im-p-AGCGA and p-AGCGA, in total 50 μM), the phosphate acceptor strand (5′-UCGCUUGCCU-3′, 50 μM), cytidine (internal standard, 200 μM), NaCl (200 mM), $MgCl_2$ (50 mM), *N*-MeIm (50 mM) and HEPES (50 mM, pH 8), was incubated at 20 °C.

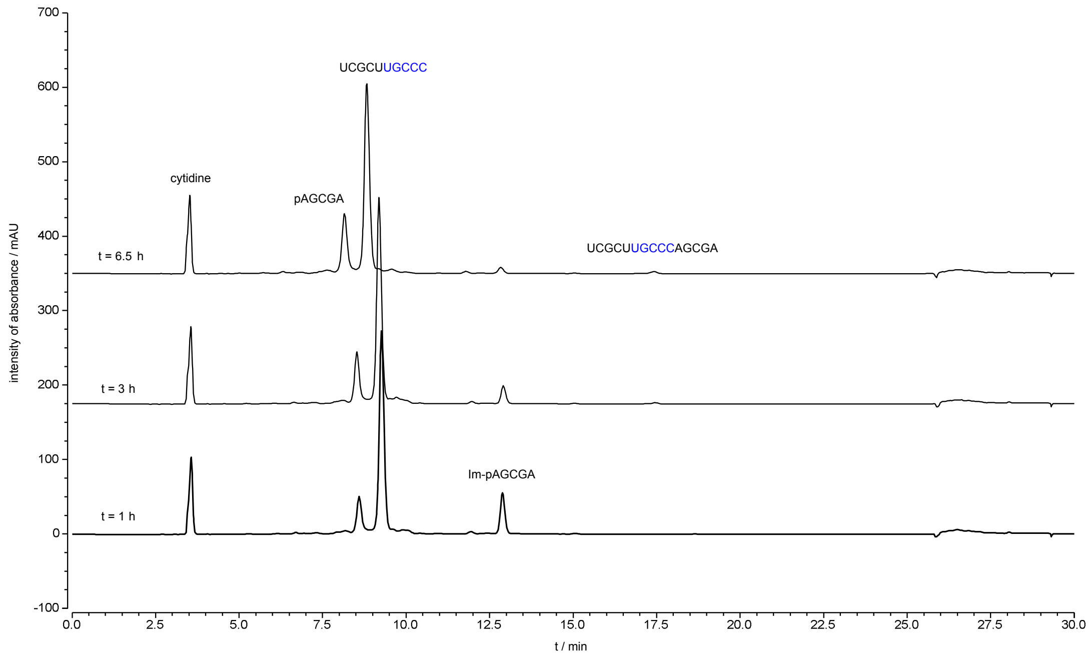

Figure S15. Stacked HPLC traces of loop-closing ligation with UGCCC overhang.

Loop duplex sequence:

`3'AGCGAp-Im`

`5'UCGCUUGCCC`

Loop-closing ligation was monitored by HPLC with 260 nm UV detection. The solution was incubated at 20 °C and aliquots of 8 µL were injected into an HPLC at different time points. Peaks for the phosphate donor, phosphate acceptor strands and the product of loop-closing ligation are indicated. Conditions: 50 µL of reaction mixture, containing the phosphate donor strand (including Im-p-AGCGA and p-AGCGA, in total 50 µM), the phosphate acceptor strand (5′-UCGCUUGCCC-3′, 50 µM), cytidine (internal standard, 200 µM), NaCl (200 mM), $MgCl_2$ (50 mM), *N*-MeIm (50 mM) and HEPES (50 mM, pH 8), was incubated at 20 °C.

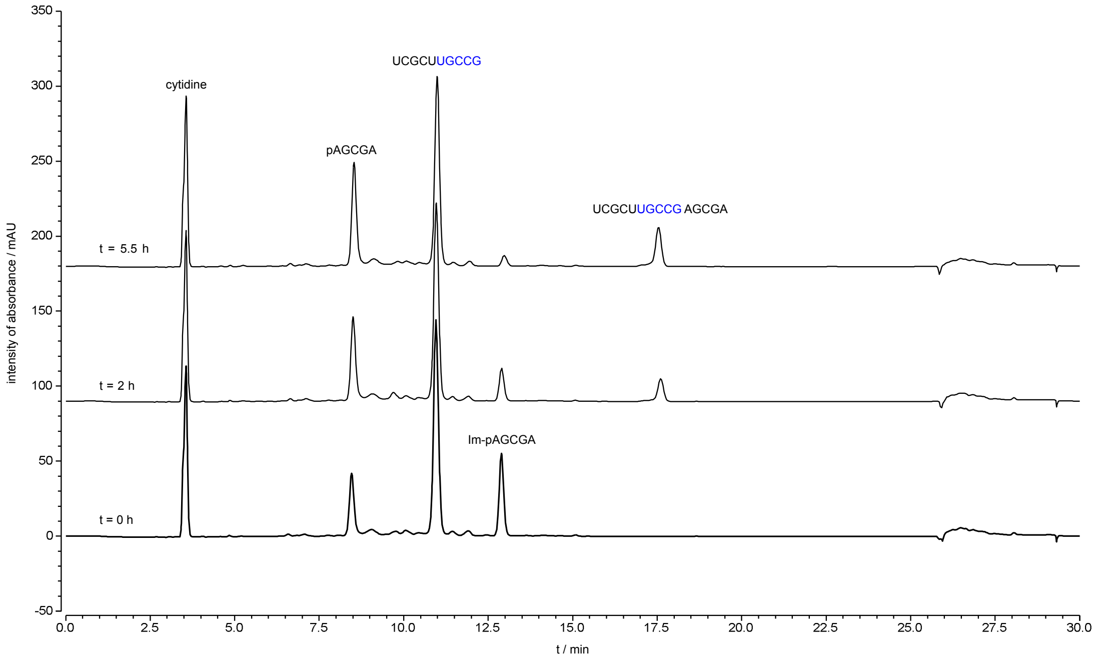

Figure S16. Stacked HPLC traces of loop-closing ligation with UGCCG overhang.

Loop duplex sequence:

`3'AGCGAp-Im`

`5'UCGCUUGCCG`

Loop-closing ligation was monitored by HPLC with 260 nm UV detection. The solution was incubated at 20 °C and aliquots of 8 μL were injected into an HPLC at different time points. Peaks for the phosphate donor, phosphate acceptor strands and the product of loop-closing ligation are indicated. Conditions: 50 μL of reaction mixture, containing the phosphate donor strand (including Im-p-AGCGA and p-AGCGA, in total 50 μM), the phosphate acceptor strand (5′-UCGCUUGCCG-3′, 50 μM), cytidine (internal standard, 200 μM), NaCl (200 mM), $MgCl_2$ (50 mM), *N*-MeIm (50 mM) and HEPES (50 mM, pH 8), was incubated at 20 °C.

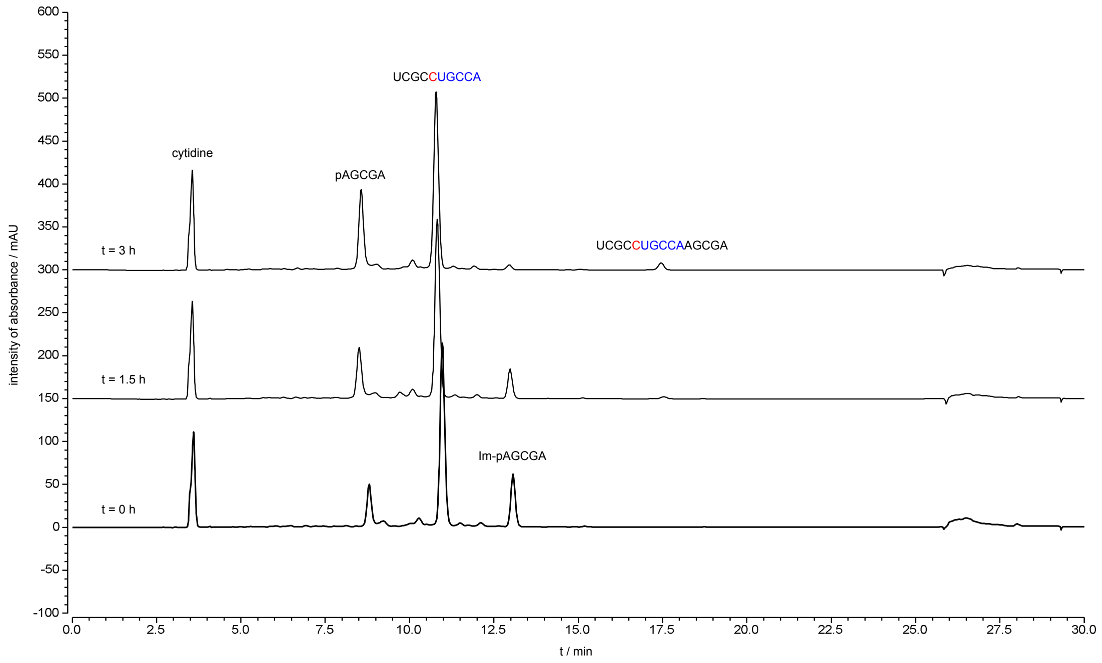

Figure S17. Stacked HPLC traces of loop-closing ligation with CUGCCA overhang on 3'-end and A on 5'-end. Loop duplex sequence:

`3'AGCGAp-Im`

`5'UCGCCUGCCA`

Loop-closing ligation was monitored by HPLC with 260 nm UV detection. The solution was incubated at 20 °C and aliquots of 8 µL were injected into an HPLC at different time points. Peaks for the phosphate donor, phosphate acceptor strands and the product of loop-closing ligation are indicated. Conditions: 50 µL of reaction mixture, containing the phosphate donor strand (including Im-p-AGCGA and p-AGCGA, in total 50 µM), the phosphate acceptor strand (5′-UCGCCUGCCA-3′, 50 µM), cytidine (internal standard, 200 µM), NaCl (200 mM), $MgCl_2$ (50 mM), *N*-MeIm (50 mM) and HEPES (50 mM, pH 8), was incubated at 20 °C.

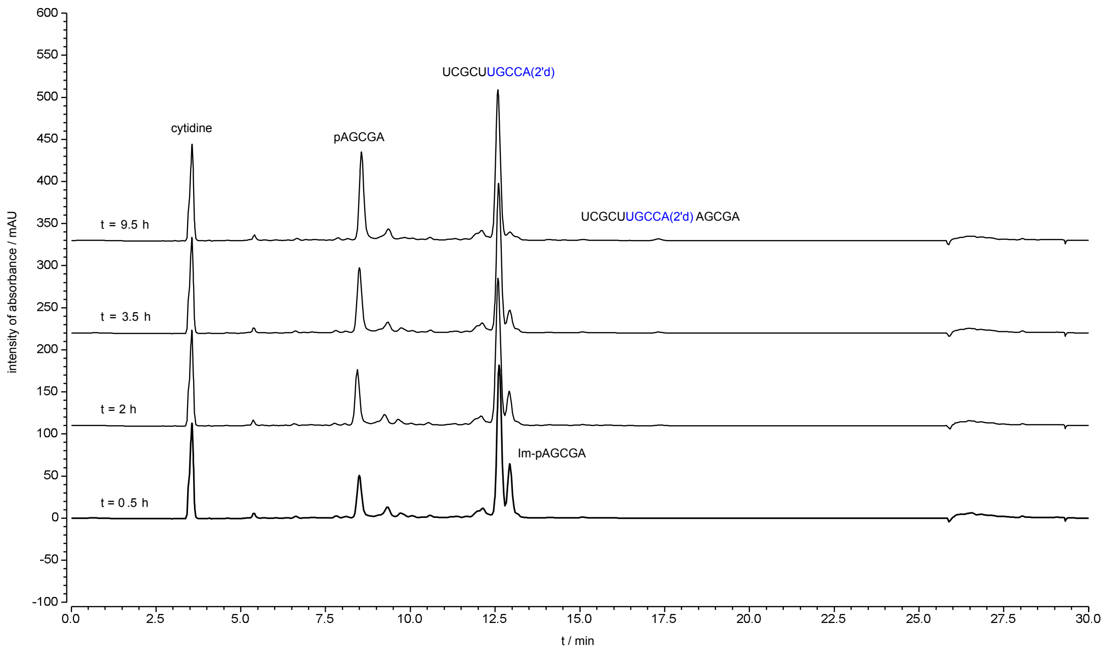

Figure S18. Stacked HPLC traces of loop-closing ligation with UGCCA(2'd) overhang.

Loop duplex sequence:

3′AGCGAp-Im

5′UCGCUUGCCA(2′d)

Loop-closing ligation was monitored by HPLC with 260 nm UV detection. The solution was incubated at 20 °C and aliquots of 8 µL were injected into an HPLC at different time points. Peaks for the phosphate donor, phosphate acceptor strands and the product of loop-closing ligation are indicated. Conditions: 50 µL of reaction mixture, containing the phosphate donor strand (including Im-p-AGCGA and p-AGCGA, in total 50 µM), the phosphate acceptor strand (5′-UCGCUUGCCA(2′d)-3′, 50 µM), cytidine (internal standard, 200 µM), NaCl (200 mM), $MgCl_2$ (50 mM), *N*-MeIm (50 mM) and HEPES (50 mM, pH 8), was incubated at 20 °C.

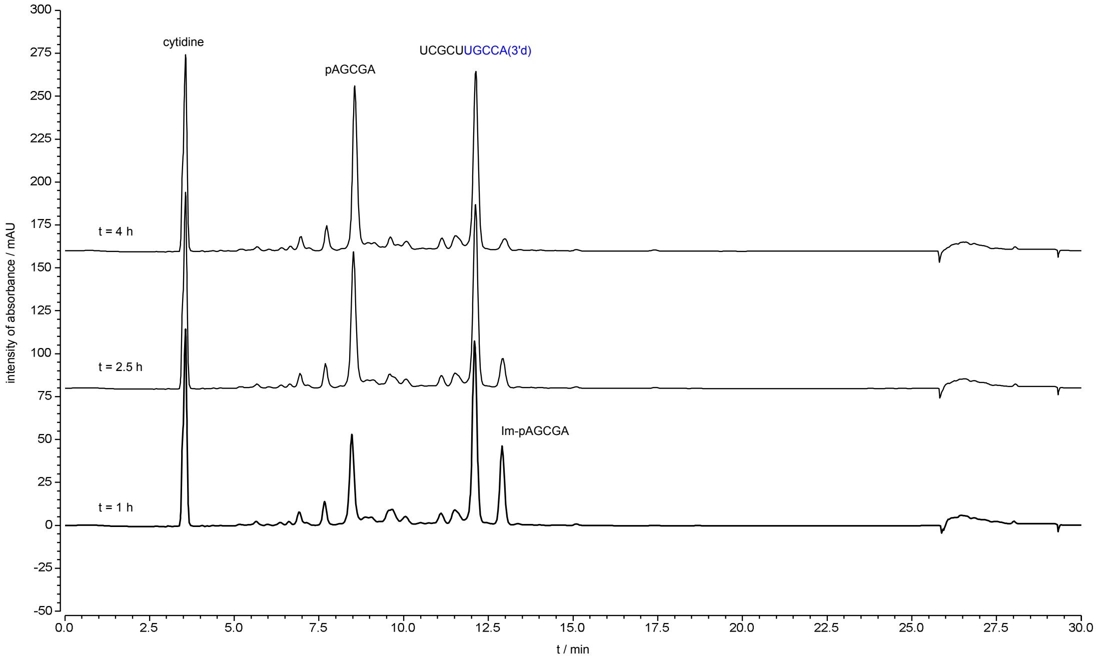

Figure S19. Stacked HPLC traces of loop-closing ligation with UGCCA(3'd) overhang.

Loop duplex sequence:

```
3'AGCGAp-Im
5'UCGCUUGCCA(3'd)
```

Loop-closing ligation was monitored by HPLC with 260 nm UV detection. The solution was incubated at 20 °C and aliquots of 8 μL were injected into an HPLC at different time points. Peaks for the phosphate donor, phosphate acceptor strands and the product of loop-closing ligation are indicated. Conditions: 50 μL of reaction mixture, containing the phosphate donor strand (including Im-p-AGCGA and p-AGCGA, in total 50 μM), the phosphate acceptor strand (5′-UCGCUUGCCA(3′d)-3′, 50 μM), cytidine (internal standard, 200 μM), NaCl (200 mM), $MgCl_2$ (50 mM), *N*-MeIm (50 mM) and HEPES (50 mM, pH 8), was incubated at 20 °C.

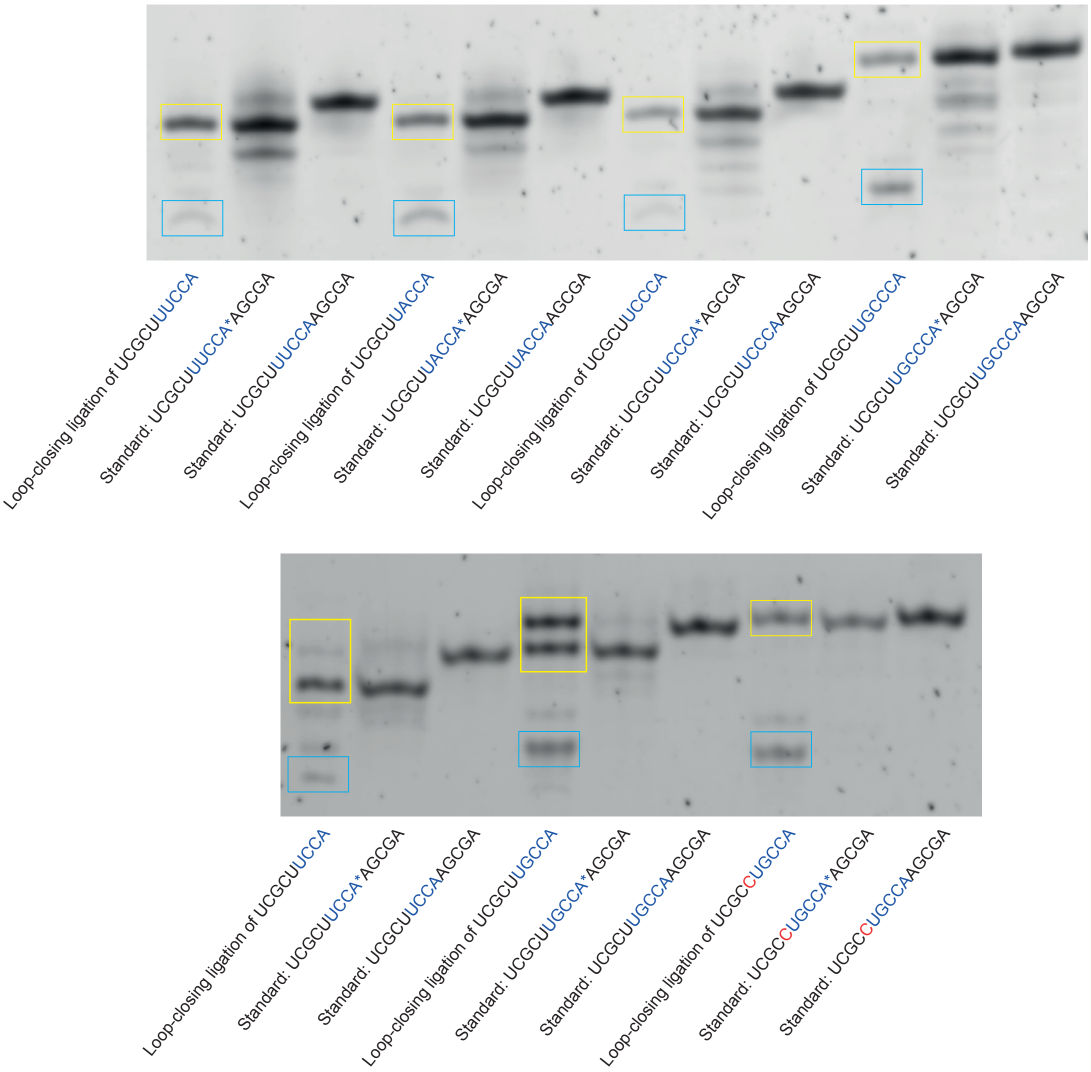

Figure S20. Characterisation of the regioselectivity of loop-closing ligations by PAGE. Starting materials (9 mer to 11 mer, Table 1) are indicated in blue boxes. Loop-closing ligation products are indicated in yellow boxes. A synthesised all- 3′-5′-linkage authentic standard and an authentic standard with one 2′-5′-linkage at the loop-closing position were both run in parallel on the gel for comparison. A*A indicates a 2′-5′-linkage between these two nucleosides. The gel was stained by using SYBR Gold Nucleic Acid Gel Stain (no dye-labelling of those oligos) before imaging. The newly formed phosphodiester bond was predominantly 2′-5′-linked for the first five reactions, but predominantly 3′-5′-linked for the last two reactions.

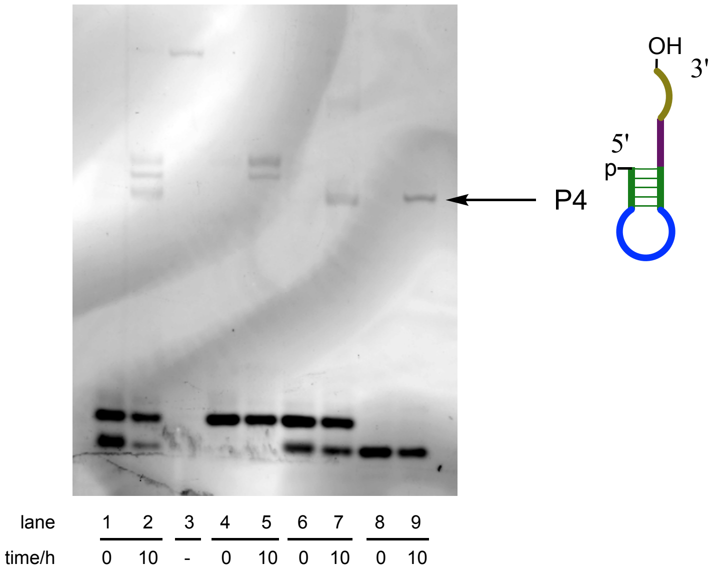

Figure S21. Direct assembly a minihelix RNA structure by loop-closing ligation (SYBR gold staining of the RNA gel shown in Figure 2). Lane 1&2, assembly reaction of RNA-1, Im-p-RNA-2 and Im-p-RNA-3; Lane 3, authentic standard of the minihelix RNA; Lane 4&5, reaction of RNA-1 and Im-p-RNA-3; Lane 6&7, reaction of RNA-1, Im-p-RNA-2 and p-RNA-3; Lane 8&9, reaction of p-RNA-2 and Im-p-RNA-3.

A) Assembly of a full-length ligase and the enzymatic ligation reaction

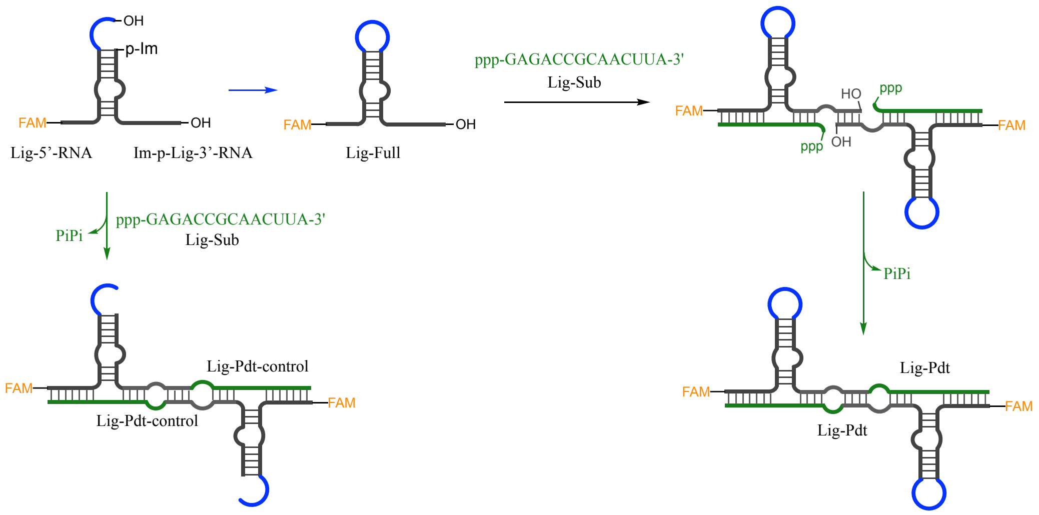

B) PAGE results of assembly and enzymatic assay reactions

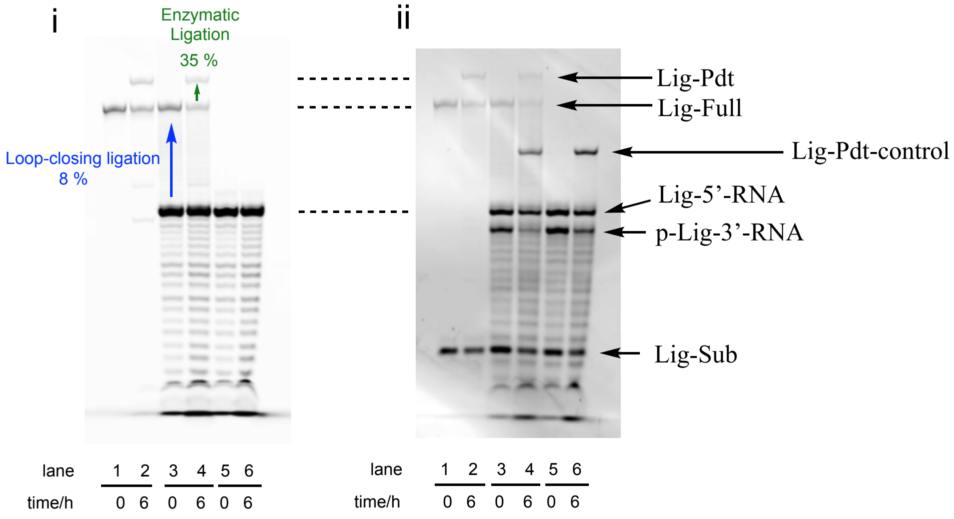

Figure S22. Direct assembly of the Joyce ligase ribozyme and the enzymatic ligation assay. A) Reaction scheme of the assembly of the ribozyme ligase and its subsequent enzymatic reaction. B) Representative PAGE gel electrophoresis for the assembly reaction and the enzymatic assay. i) Imaging based on FAM-labelling; ii) Imaging the same gel after SYBR-gold staining. Lane 1-2, positive control reaction of the Joyce ligase by using a pre-synthesised full-length ribozyme; Lane 3-4, enzymatic ligation reaction after loop-closing ligation; Lane 5-6, negative control without preceding loop-closing ligation.

Phosphate donor	Phosphate acceptor sequence		pH	Temperature	NaCl (mM)	$MgCl_2$ (mM)	*N*-MeIm (mM)	Observed yield	Corrected yield	Reaction half-life (h)
	Stem	Overhang								
Im-p-AGCGA	UCGCU	UGCCA	8.0	20 °C	200	50	0	9 %	16 %	40
			8.0	20 °C	200	50	10	18 %	30 %	7.5
			8.0	20 °C	200	50	20	17 %	28 %	3.3
			8.0	20 °C	200	50	50	18 %	30 %	1.8
			8.0	20 °C	200	50	80	18 %	30 %	1.3
			8.0	20 °C	200	50	100	18 %	30 %	1.0
			8.0	20 °C	200	50	200	17 %	28 %	0.5
			8.0	30 °C	200	50	50	16 %	27 %	--
			8.0	4 °C	200	50	50	27 %	45 %	--

Table S1. The reaction rates of loop-closing ligation depend on the concentration of *N*-methylimidazole (*N*-MeIm). The blue colour highlights the reference condition. Corrected yield = Observed yield divided by the initial fraction of Im-p-AGCGA present in the pre-synthesized mixture of Im-p-AGCGA & p-AGCGA (for synthetic methods see the SI). Reaction half-life, $t_{1/2}$, is the combined rate of first-order consumption of Im-p-AGCGA resulting from both loop-closing ligation and the competing hydrolysis. All yields and half-lives are average values from at least two independent experiments.

Phosphate donor	Phosphate acceptor sequence		pH	Temperature	NaCl (mM)	$MgCl_2$ (mM)	*N*-MeIm (mM)	Observed yield	Corrected yield	Reaction half-life (h)
	Stem	Overhang								
Im-p-AGCGA	UCGCU	UGCCA	5.2	20 °C	200	50	50	0 %	0 %	0.4
			6.0	20 °C	200	50	50	< 1 %	<1 %	0.5
			7.0	20 °C	200	50	50	5 %	8 %	0.5
			7.5	20 °C	200	50	50	12 %	18 %	0.6
			8.0	20 °C	200	50	50	18 %	30 %	1.8
			9.2	20 °C	200	50	50	16 %	27 %	6.7

Table S2. The pH-dependence of the loop-closing ligation. The blue colour highlights the reference condition. All yields and half-lives are average values from at least two independent experiments.

Phosphate donor	Phosphate acceptor sequence		pH	Temperature	NaCl (mM)	$MgCl_2$ (mM)	*N*-MeIm (mM)	Observed yield	Corrected yield	Reaction half-life (h)
	Stem	Overhang								
Im-p-AGCGA	UCGCU	UGCCA	8.0	20 °C	200	0	50	2 %	3 %	0.8
			8.0	20 °C	200	10	50	6 %	10 %	1.1
			8.0	20 °C	200	20	50	13 %	21 %	1.4
			8.0	20 °C	200	50	50	19 %	31 %	1.8
			8.0	20 °C	200	100	50	25 %	41 %	2.4
			8.0	20 °C	200	200	50	29 %	48 %	2.9
			8.0	20 °C	200	500	50	29 %	48 %	4.9

Table S3. The yields of loop-closing ligation depend on concentration of $MgCl_2$. The blue colour highlights the reference condition. All yields and half-lives are average values from at least two independent experiments.

Phosphate Donor	Phosphate acceptor sequence		pH	Temperature	NaCl (mM)	$MgCl_2$ (mM)	*N*-MeIm (mM)	Observed yield	Corrected yield	Reaction half-life (h)
	Stem	Overhang								
Im-p-AGCGA	UCGCU	UGCCA	8.0	20 °C	0	0	50	1 %	2 %	0.4
			8.0	20 °C	100	0	50	3 %	4 %	0.7
			8.0	20 °C	200	0	50	4 %	6 %	1.0
			8.0	20 °C	500	0	50	5 %	10 %	1.5
			8.0	20 °C	1000	0	50	6 %	15 %	2.2
			8.0	20 °C	2000	0	50	16 %	22 %	3.9

Table S4. The yields of loop-closing ligation depend on concentration of NaCl. The blue colour highlights the reference condition. All yields and half-lives are average values from at least two independent experiments.
